# Classification of HIV-1 Sequences Using Profile Hidden Markov Models

**DOI:** 10.1371/journal.pone.0036566

**Published:** 2012-05-18

**Authors:** Sanjiv K. Dwivedi, Supratim Sengupta

**Affiliations:** 1 School of Computational and Integrative Sciences, Jawaharlal Nehru University, New Delhi, India; 2 School of Sciences, Indian Institute of Technology, Indore, Indore, India; 3 Department of Physical Sciences, Indian Institute of Science Education and Research, Kolkata, Mohanpur, West Bengal, India; British Columbia Centre for Excellence in HIV/AIDS, Canada

## Abstract

Accurate classification of HIV-1 subtypes is essential for studying the dynamic spatial distribution pattern of HIV-1 subtypes and also for developing effective methods of treatment that can be targeted to attack specific subtypes. We propose a classification method based on profile Hidden Markov Model that can accurately identify an unknown strain. We show that a standard method that relies on the construction of a positive training set only, to capture unique features associated with a particular subtype, can accurately classify sequences belonging to all subtypes except B and D. We point out the drawbacks of the standard method; namely, an arbitrary choice of threshold to distinguish between true positives and true negatives, and the inability to discriminate between closely related subtypes. We then propose an improved classification method based on construction of a positive as well as a negative training set to improve discriminating ability between closely related subtypes like B and D. Finally, we show how the improved method can be used to accurately determine the subtype composition of Common Recombinant Forms of the virus that are made up of two or more subtypes. Our method provides a simple and highly accurate alternative to other classification methods and will be useful in accurately annotating newly sequenced HIV-1 strains.

## Introduction

HIV affects millions of people worldwide and poses significant public health challenges especially in developing countries which lack the resources to effectively combat the AIDS pandemic. A major characteristic of HIV virus is its ability to rapidly mutate into different forms thereby eluding the ability of drugs to effectively attack and destroy it. While a complete cure for HIV/AIDS still eludes researchers, more recently a cocktail of drugs has proved quite effective in considerably enhancing the life-span of patients [1] infected by the virus. However, the drug cocktails have to be designed to target a specific strain of virus and it is therefore essential to know the strain which infects the patient in order to prescribe an effective course of treatment.

HIV-1 strains have been classified into three distinct groups – M (major), O (outlier) and N (non-M, non-O) based on their genetic variability. Most HIV-1 viruses fall in group M which has been further sub-divided [2], [3] into distinct subtypes – A,B,C,D,F,G,H,J,K based on phylogenetic clustering of sequences. Some subtypes like A (and F) have been further sub-divided into sub-subtypes A1, A2 (and F1, F2) also based on phylogenetic clustering of sequences within the subtype.

Analysis of the geographical spread of different HIV strains [4] indicates that subtype C is found predominantly in China, India, Nepal and South Africa; while subtype B is most commonly found in patients residing in the Americas, Australia, Japan, Thailand and Western Europe. Subtypes A and D are common in Africa while subtype F has been found predominantly in Central Africa, South America and Eastern Europe. However, the ease of trans-continental mobility implies that patients infected by a strain not typically found in a particular region can often appear in that region [5], [6]. This has important implications for treatment since many retroviral drug cocktails are specifically tailored for a particular strain. It is therefore essential to develop an efficient method for subtype determination of an HIV-1 strain. Developing effective treatments against HIV-1 is further complicated by the presence of Circulating Recombinant Forms (CRFs) which frequently arise as a result of recombination [7] of two or more HIV-1 subtypes. This poses the additional challenge of effectively determining the subtype composition of a CRF.

Most of the methods developed so far to determine the subtype of unknown HIV-1 sequences are based on obtaining pairwise-distances between sequences or on phylogenetic analysis [8]–[14]. In these methods subtype determination was based on the clustering of the unknown sequence in the phylogenetic tree with sequences of a known subtype. Early work on classification [8] made use of the genetic variation of the *env* region of HIV-1 sequences to construct the phylogenetic tree. Subtypes A to F could be successfully classified using this method. The gag region of HIV-1 was also used [9] for phylogenetic tree construction and improved classification. Phylogenetic analysis based on both *env* and gag regions [10]–[12] were subsequently used to reveal the presence of subtypes G to J. However, phylogenetic studies based on these regions alone were unable to distinguish between distinct clusters within subtypes A and F which were later found from phylogenetic tree construction based on whole-genome analysis [13]. Further investigations [14] also revealed earlier errors in classification [11] by pointing out that subtype I was found to be a CRF made up of subtypes A and G. It has become clear that phylogenetic analysis based on complete genomes is much more reliable than those based on short segments of the HIV-1 genome. However, complete genomes are often not available and there is need for a method which accurately determines the subtype of strains for which only a segment of the genome has been sequenced.

More recently several new methods [15]–[20] have been developed to improve the accuracy of HIV-1 subtype classification. Gale *et al.* [16] and Myers *et al.* [17] developed a classification tool (STAR) that used subtype specific profiles created by Position Specific Scoring Matrices (PSSMs) from multiple sequence alignments of HIV-1 sequence data. These subtype profiles were then used to score an unknown sequence to determine its subtype with high accuracy. Hraber *et al.* [18] developed a method that combines information from pairwise distances and phylogenetic methods. They used the branching index (BI) to determine how closely a test sequence clusters with sequences belonging to a given subtype. Pandit *et al.* [19] used Chaos Game Representation to find subtype specific patterns which allow for accurate classification.

Hidden Markov Models (HMMs) have been successfully used [21]–[23] for identifying conserved patterns in protein [24] and nucleotide [25] sequences. Profile HMMs (pHMMs) are constructed from multiple sequence alignments (MSA) of a set of homologous sequences and can capture the unique characteristics of the set. Hence they can be used to determine whether an unknown sequence can be considered to be homologous to the set of sequences that were used to build the MSA. Recently, pHMMs have also been shown [25] to be extremely useful for fast and accurate classification of a type of regulatory RNA called riboswitches. Variations of pHMMs have also been used to detect breakpoints in recombinant HIV-1 strains by several research groups [26]–[30].

Recently a method based on pHMM called the jumping profile Hidden Markov Model (jpHMM) has been developed [26]–[27] to accurately determine the subtype composition as well as determine the location of breakpoints in CRFs. jpHMM is a generalization of the jumping alignment method [31] and is based on aligning a query sequence to the multiple sequence alignment of entire subtypes and not just to a single reference sequence belonging to a subtype as in the case of an the jumping alignment algorithm. Jumps between subtypes are allowed with a low probability (which is tuned) albeit with certain restrictions. Using this method, Schultz *et al.* [26]–[27] have been able to predict the subtype composition of CRFs as well determine the location of the breakpoints with higher accuracy compared to the popular recombination detection tool SimPlot [28]. Very recently, Truszkowski and Brown [29] have developed a new algorithm to improve the accuracy of breakpoint predictions by the jpHMM method. Westesson and Holmes [30] combined phylogenetic analysis and HMMs to detect breakpoints accurately by detecting changes in phylogenetic tree topology across multiple sequence alignments. All these papers focus on the accuracy of breakpoint detection in recombinant HIV-1 strains.

Our main objective in this paper is to present an efficient and highly accurate pHMM-based method to classify HIV-1 sequences. Although our method cannot detect recombination breakpoints, it nevertheless provides a simple and unified scheme for detecting the subtype composition of CRFs with high accuracy.

## Results

We have used profile Hidden Markov Models to classify sequences belonging to the M group of HIV-1 to appropriate subtypes. We find that the standard method of classification (described in the “Methods” section) based on construction of a positive training set containing six sequences known to belong to a particular subtype, is adequate for classifying the strains of HIV-1 that belong to subtypes A,C,F and G with 100% sensitivity and specificity. However, the close similarity between sequences that belong to subtypes B and D results in reduction of the accuracy of classification using the standard method. We then demonstrate the use of an improved method based on construction of a positive and a negative training set for accurately discriminating between sequences belonging to the B and D subtypes. We find that these sequences can be classified with 100% accuracy using the improved method. Finally, we demonstrate how our pHMM-based method of classification can be used to identify the composition of CRF’s that contain segments belonging to multiple subtypes of HIV-1.

### Classification using the Standard Method

**Figure 1:**
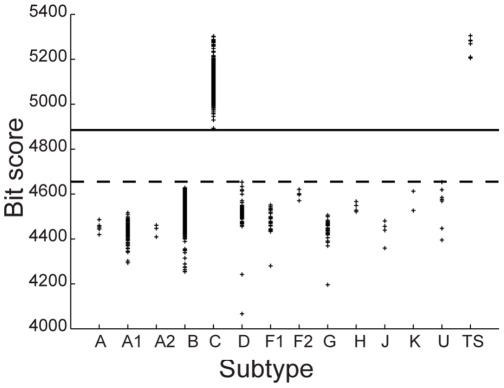
Subtype C classification using the standard method. Distribution of Z-scores for all HIV-1, group M sequences in the database when the training set (labelled TS) is constructed using six sequences belonging to the subtype C. Tp (dashed line) denotes the minimum score of some sequences belonging to the test set and Tn (solid line) denotes the maximum score of the remaining sequences.

**Figure 2:**
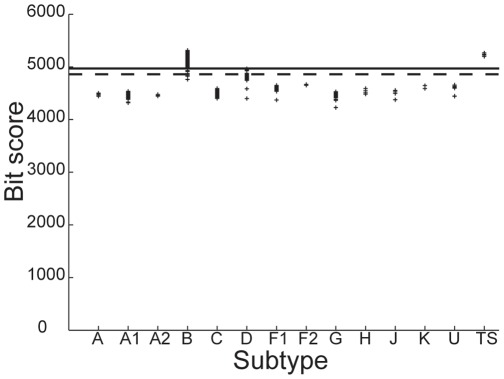
Subtype B classification using the standard method. Distribution of Z-scores for all HIV-1 group M sequences in the database when the training set is constructed using sequences belonging to the subtype B.

A pHMM is constructed for each HIV-1 subtype using a positive training set only. It is then used to determine the bit-scores of all sequences belonging to the test set. For a given threshold, which is arbitrarily fixed for a given subtype, based on the distribution of scores for all the sequences in the test-set; the model can predict whether the query sequence can be considered to be a member of that subtype, based on its bit score. Fig. 1, 2, 3 below shows the distribution of scores of all the sequences belonging to group M of HIV-1 when the training sets are constructed using sequences known to belong to subtypes C, B and D respectively.

When sequences belonging to subtype C are used to construct the training set, a clear demarcation is observed (Fig.1) between the minimum score of some sequences from the test set (indicated by Tp) and the maximum score of the remaining sub-set (indicated by Tn). The former sequences are found to belong to subtype C and the latter sequences belong to subtypes other than C. Hence specifying a threshold between Tp and Tn allows for classification of sequences belonging to the C subtype with 100% accuracy.

**Figure 3:**
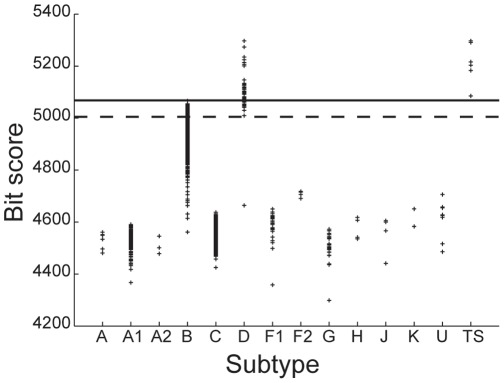
Subtype D classification using the standard method. Distribution of Z-scores for all HIV-1 group M sequences in the database when the training set is constructed using sequences belonging to the subtype D.

Similar results are obtained for classification of sequences that belong to subtypes of M other than B and D. However, the standard method was unable to accurately discriminate between sequences belonging to the B and D subtypes. This is evident from Fig. 2 and 3 which show the Z-score distributions that is obtained when sequences from subtypes B and D respectively are used to construct the training set. Both the figures show significant overlap between the scores of some of the sequences that belong to B and D subtypes. Therefore an appropriate threshold cannot be found for which all sequences belonging to B and D subtypes can be accurately classified. These results indicate a close similarity between strains B and D as a result of which the profile obtained on the basis of the positive training set of either B or D only is insufficient to capture the unique characteristics of strains B and D.

### Performance of Models Constructed for HIV-1 Subtypes Belonging to the Group M

**Figure 4:**
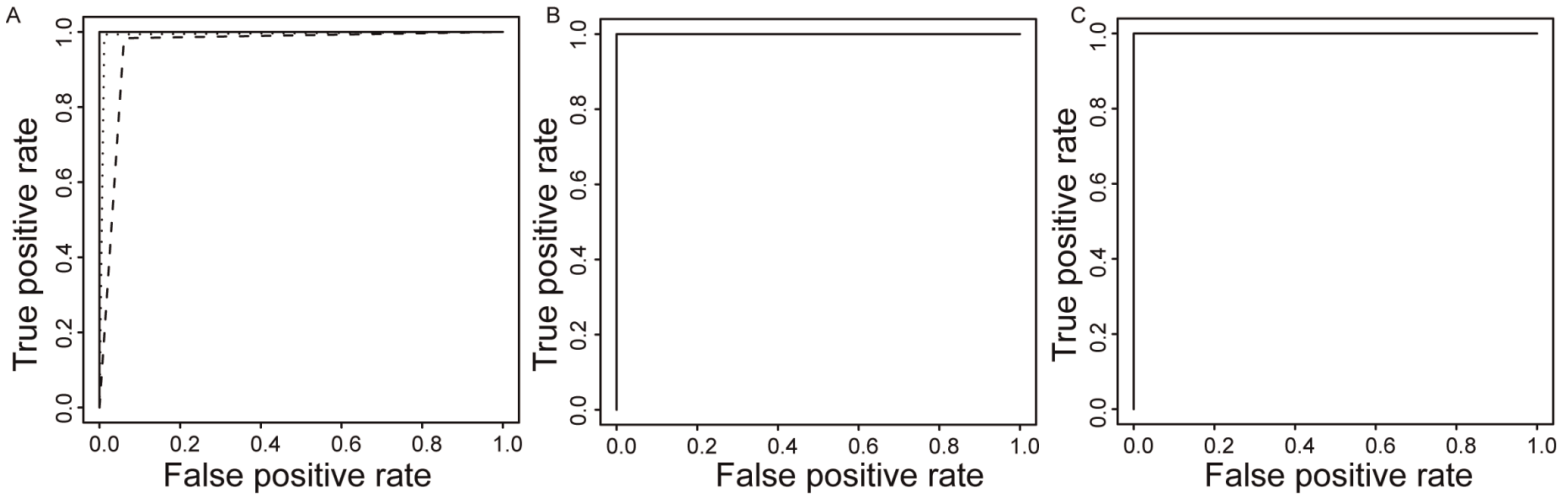
ROC curves. (a) ROC curves for subtypes C (solid line), B (dotted line) and D (dashed line) obtained using the standard method. Roc curves for (b) B, (c) D; obtained using the improved method.

To determine the effectiveness of our pHMMs in accurately classifying query sequences into appropriate subtypes, it is necessary to determine the sensitivity and specificity of our profile Hidden Markov Models. For a fixed threshold score, the pHMMs can predict whether a specific query sequence in the test-set is a true positive (TP) if it correctly identifies it as belonging to the specific subtype for which the model was constructed or a false negative (FN) if it incorrectly identifies it as a non-member. Similarly, the model can correctly classify non-members as true negatives (TN) or false positives (FP) when it incorrectly predicts a non-member to be a homolog. Sensitivity and Specificity are then defined as Sensitivity = TP/(TP+FN) and Specificity = TP/(TP+FP). The Receiver Operator Characteristic (ROC) Curves obtained by plotting Sensitivity v/s 1-Specificity is a measure of the ability of each pHMM to accurately classify the test sequence and discriminate between sequences belonging to different subtypes. ROC curves for subtypes C, B and D are shown in Fig.4. The plot shown in Fig. 4(a) is obtained using the standard method while the ones in Fig. 4(b) and 4(c) have been obtained using the improved method. Fig. 4(a) clearly indicates that the pHMM for subtype C is able to classify sequences with 100% sensitivity and specificity. pHMM’s for all subtypes except B and D also show perfect sensitivity and specificity. The standard method of classification using pHMMs is not able to discriminate between sequences belonging to subtypes B and D with 100% accuracy as evident from dotted and dashed lines in Fig. 4(a). However, when the improved method of classification is used, performance of the pHMM classifier improves significantly and it is able to accurately classify sequences belonging to B and D subtypes as indicated by the ROC curves in Fig. 4(b) and Fig.4(c).

### Classification using the Improved Method

The standard method of classification while effective in some cases, suffers from two main drawbacks. Firstly it is unable to accurately discriminate between distinct strains which nevertheless show close sequence similarity and secondly, the threshold for distinguishing true positives from true negatives has to be arbitrarily chosen. The threshold can change as the number of sequences in the database changes. To avoid these problems, we used the improved method of classification using pHMMs. In this improved method, pHMMs built from a positive training set and a negative training set are associated with each subtype. Using both positive and negative training sets allow for construction of models that are finely tuned to identify the unique features of a specific subtype and detect small differences between strains that belong to closely related subtypes. Moreover, the threshold for distinguishing true positives from false positives can be unambiguously fixed to a Z-score of zero in this method. Using this method, it is even possible to distinguish between sub-subtypes F1 and F2 of subtype F, (See Fig.S3 in “Supporting information”) as well as sub-subtypes A1 and A2 of subtype A (data not shown).

**Figure 5:**
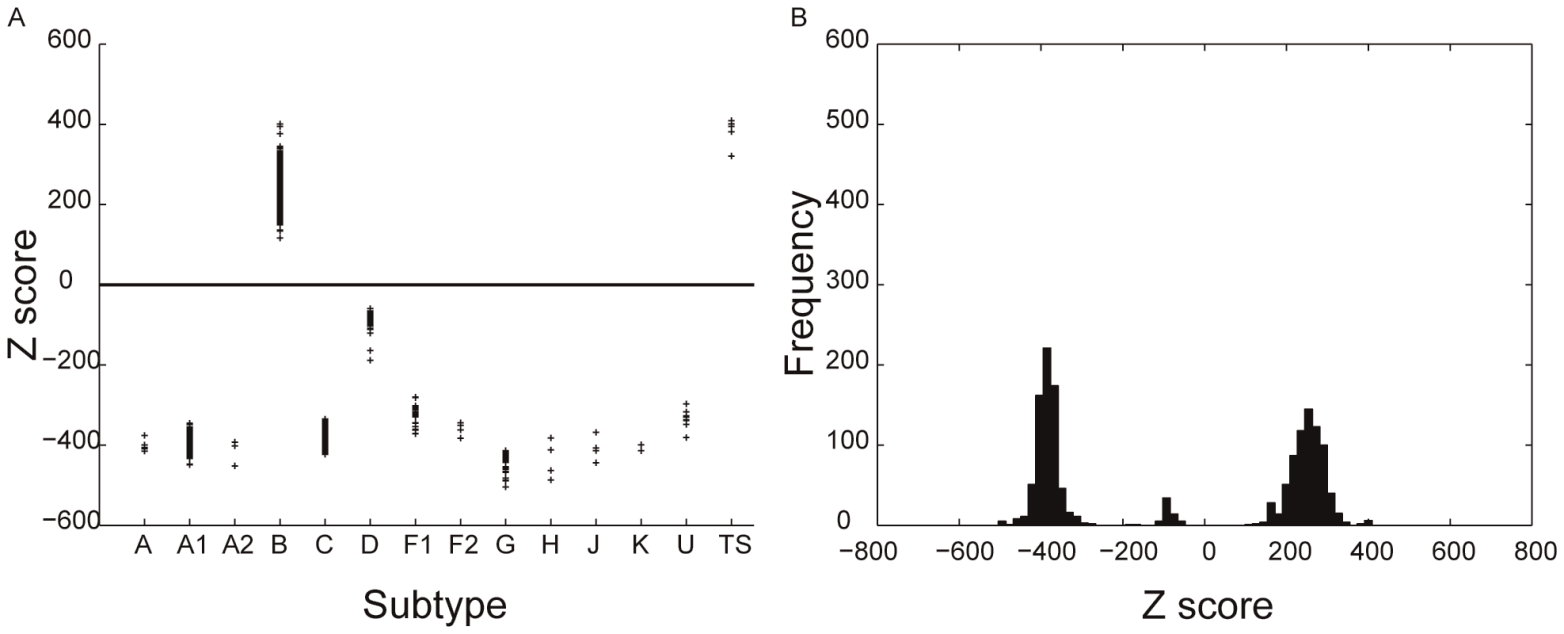
Subtype B classification using the improved method. (a) Distribution of Z-scores for group M sequences; (b) Frequency distribution of Z-scores, when six sequences of subtype B are used in the positive training set.

**Figure 6:**
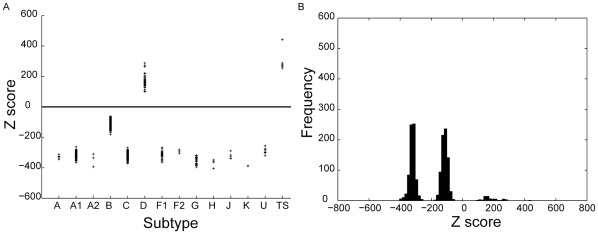
Subtype D classification using the improved method. (a) Distribution of Z-scores for the test and training sets; (b) Frequency distribution of Z-scores, when six sequences of subtype D are used in the positive training set.

Fig.5–6 shows the distribution of Z-scores for sequences of the test set as well as the training set when the profile HMM is built for subtypes B and D respectively. The X-axis shows the subtypes to which the strains in the test set belong, as annotated in the Los Alamos HIV database. In Fig.5, the strains belonging to subtype B are clearly distinguished from all other strains in the test set by a positive Z-score. The improved method also leads to classification of sequences belonging to subtype D (Fig. 6) with 100% accuracy. This is also evident from the histogram plot (Fig.5b, 6b) showing the Z-score frequency distribution. However, in the case of D classification, this is achieved only after increasing the number of B subtype sequences in the negative training set from two to six to allow for improved characterization of the D profile. The accession numbers of sequences used for building the positive and negative training sets are given in Tables S1, S2, S3, S4 in the “Supporting information”. Results of classification of other subtypes using the improved method are shown in Figs.S1, S2, S3, S4, S5 in the “Supporting Information”. Results of classification of the HIV-1 sequences on the basis of the *env* region are shown in Figs.S12, S13, S14, S15, S16, S17, S18, S19 in the “Supporting information”. See Tables S7, S8, S9, S10, S11 for information on accession numbers of the sequences used to build the positive and negative training sets.

### Analysis of CRF’s

CRF’s present a unique challenge to classification methods because they are composed of two or more HIV-1 strains. The improved pHMM method described in the previous section allows us to accurately determine the mixture of subtypes that make up the CRF. The reliability of this method depends on the length of the segment of a particular subtype that is present in the region used for constructing the pHMMs. We show below how the pHMM method can be successfully employed in determining the presence of subtypes in the *gag-pol* region. If the segment length of a specific subtype in the *gag-pol* region is significant enough for its features to be captured in the pHMM, then that aspect will be reflected in the Z-score returned by the classifier. By using the pHMMs for the different subtypes to score a query CRF sequence, it is possible to identify the subtype composition by the Z-scores that are returned.

**Figure 7:**
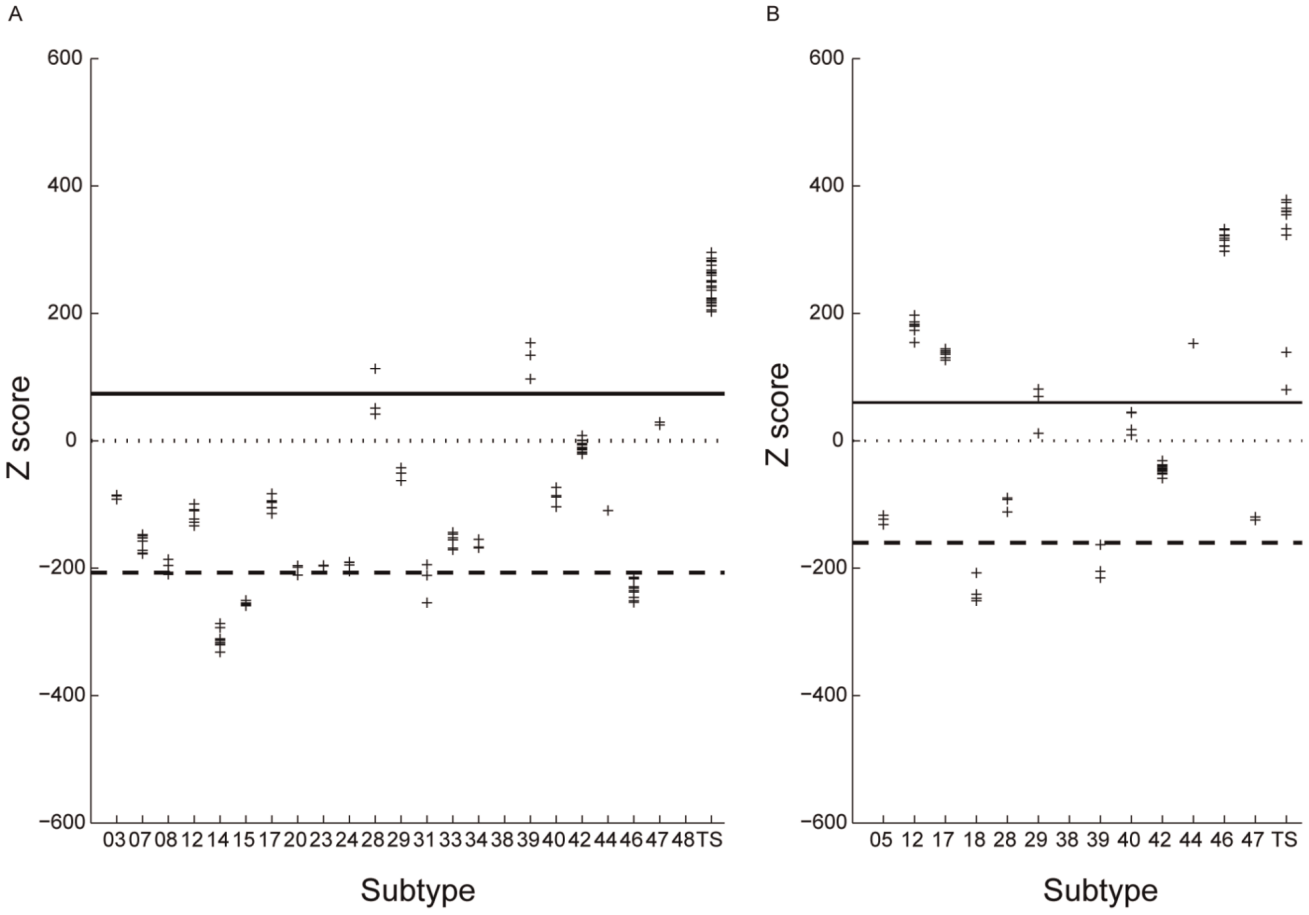
Detection of subtypes B and F in the *gag-pol* region of CRFs . Z-scores of CRF strains containing segments of (a) B subtype; (b) F subtype. The numbers on the X-axis correspond to the various CRF strains as annotated in the Los Alamos HIV database. An abbreviated annotation is used. For example, the label 14 refers to CRF14_BG and 28 refer to CRF28_BF etc. The solid and dashed lines correspond to the thresholds Tp and Tn respectively.

In the examples below, we use our method to detect CRF strains made up of B and F subtypes in the *gag-pol* region. We first determine the Z-scores of all the CRF strains in the database by using a positive classifier (pHMM) constructed using 31 sequences from subtype B and a negative classifier constructed using 2 sequences each from all subtypes *except* D for which case 22 sequences were used. This was done to enable better discrimination between B and D subtype segments. (The tables giving the number of sequences used to build the positive and negative training sets necessary for determining the subtype composition of CRFs are Tables S5 and S6 in the “Supporting information”.) Fig. 7(a) shows the Z-scores of all the CRF strains that contain one or more segments of B subtype as specified in the Los Alamos HIV1 database. The X-axis shows the CRF strains (containing B subtype) that are listed in the database and the Y-axis gives the corresponding Z-scores. In two (CRF28_BF and CRF_39BF) out of the twenty-three instances, the sequences have Z-scores that are either greater than or very close to the threshold Tp. In these cases, most of the *gag-pol* segment consists of B subtype which explains the high Z-scores. In three (CRF14_BG, CRF15_01B, CRF46_BF) out of the twenty-three instances, the CRF strains containing B have Z-scores below Tn. In all these cases, the *gag-pol* segment does not contain any portion belonging to the B subtype. For example, in the case of CRF46_BF, the *gag-pol* segment consists entirely of subtype F and subtype B is present only in the *nef* segment and in the 3′ LTR. [32]. For strains whose Z-scores are very close to the threshold Tn (CRF_08BC, CRF_20BG, CRF_23BG, CRF_24BG, CRF_31BC), only a small region of B is present in the *gag-pol* segment. For example, in the case of CRF_31BC, the B subtype region has a length of approximately 223 nucleotides [33]. Even in these cases, one of the three sequences belonging to these strains has a Z-score above the threshold Tn and two sequences have Z-scores below but close to the threshold Tn. This is also consistent with the subtype prediction of the jpHMM method. From this analysis we conclude that sections belonging to B subtype are present in the *gag-pol* segment of only twenty out of the twenty-three CRF strains containing B. To avoid cluttering the figure, we have not shown the Z-scores for CRF strains that do not contain the B-subtype. Their Z-scores fall below the Tn threshold.

We then determined the Z-scores of all the CRF strains in the database by using a positive classifier (pHMM) constructed with using 10 sequences from subtype F (8 of F1 and 2 of F2) and a negative classifier constructed using 2 sequences each from all subtypes *except* F1 and F2. Fig. 7(b) shows the Z-scores of all the CRF strains that contain one or more segments of F subtype as specified in the Los Alamos HIV database. The X-axis shows the CRF strains (containing F subtype) that are listed in the database and the Y-axis gives the corresponding Z-scores. In four out of the twelve instances (CRF12_BF, CRF17_BF, CRF44_BF and CRF_46BF), the CRF strains have Z-scores that are either greater than or very close to the threshold Tp. In these cases, most or all (CRF46_BF) of the *gag-pol* segment consists of F subtype. In two instances (CRF18_cpx, CRF39_BF) instance, the CRF strains containing F have Z-scores slightly below Tn. According to the listing in the HIV database, the *gag-pol* segment in CRF18_cpx contains only a small fragment (411 nucleotides) belonging to the F subtype. However, neither our method nor the jpHMM method is able to identify this short region of subtype F. The jpHMM method detects the subtypes A,B,C,G,J in the gag-pol region while our method detects the subtypes A,G (see Figures 7(b), S6c and S9c in the “supporting information”.) These predictions are to be contrasted with the HIV database listing based on Thomson *et al.* [34] which detects subtypes A,G,F, H in addition to an unknown subtype U in the *gag-pol* region. In the case of CRF39_BF, Guimares *et al.* [35] indicates that this strain contains two disconnected regions of subtype F (having length 185 and 218 nucleotides respectively) in addition to subtype B in the *gag-pol* region. However, neither our method nor the jpHMM or STAR methods is able to detect these short segments of subtype F and all these methods predict the entire *gag-pol* region to be made up of subtype B only.

Using our classification method we were thus able to accurately identify all the ten CRF strains which contain segments from *both* B and F subtypes in their *gag-pol* region. This analysis can be repeated using profiles of other subtypes (see Figures S6, S7, S8, S9, S10, S11 in “supporting information”) as well as other segments of the HIV genome, to determine the composition of most CRF strains with a high level of accuracy.

Table 1 gives the accuracy of subtype detection in the *gag-pol* region of CRFs using our method. The second and third columns of Table 1 give the predicted and actual number of CRFs made up of the specified subtype in the *gag-pol* region respectively. Each CRF strain can have more than one sequence and we consider a subtype to be successfully detected in a particular strain if the subtype is detected in at least one sequence belonging to that strain. Except for a few cases, a specified subtype is detected in all sequences belonging to that strain. The fourth and fifth columns specify the predicted and actual number of sequences containing a specific subtype in the *gag-pol* region. The subtype detection sensitivity for each subtype is calculated on the basis of the numbers in the fourth and fifth columns. The specificity is 100% for all subtypes since we do not detect any false positives. The few cases where our method failed to detect the presence of subtype can be attributed to the presence of very short lengths of fragments associated with that subtype. The strain CRF16_A2D was missed by our method (as well as by the STAR subtype prediction method which finds it to be a pure A subtype only) because it contains two disconnected fragments (of length 256 and 179 nucleotides) that were too short to be detected by our method. Similarly, our pHMM method (as well as the jpHMM method) was unable to identify subtype G in the *gag-pol* region of the strain CRF19_cpx because of the short length (320 nucleotides) of the G fragment. The STAR method does not even identify this sequence as a CRF, predicting it to be a pure D sequence. A small number of sequences of subtype H as well as the presence of subtype H in short fragments in a very small number of the *gag-pol* region of CRFs make its detection in CRFs unreliable. Nevertheless, we were able to detect subtype H in all sequences belonging to the strain CRF27_cpx but not in the strain CRF18_cpx. As can be seen from Table 1, the subtype detection accuracy is very high for subtypes A, C, D, G and J. In fact, in spite of the small number of available sequences of subtype J, our pHMM model was successful in detecting that subtype in CRFs with 100% accuracy. The STAR method could not identify the J and H containing CRFs, predicting them to be pure subtypes.

**Table 1:** Accuracy of subtype detection in the *gag-pol* region of CRFs.

Subtype present in the *gag-pol* region of CRFs	Predicted #CRFs containing the subtype	Actual #CRFs containing the subtype	Predicted #sequences containing subtype X	Actual #sequences containing subtype X	Sensitivity
A	23	23	409	410	99.76%
B	19	19	91	95	95.79%
C	4	4	24	24	100%
D	5	6	23	25	92%
F	11	13	54	61	88.53%
G	15	16	133	136	97.79%
H	1	2	3	7	42.86%
J	3	3	19	19	100%

We also analysed the *env* region for subtype detection. In contrast to the *gag-pol* region, the *env* region contains relatively fewer breakpoints. Moreover, in many instances it contains only a very short fragment (<200 nucleotides) of one of the two subtypes making up the *env* region. Hence the subtype detection accuracy in the *env* region is relatively lower for both our method as well as the jpHMM method. Figures S20, S21, S22, S23, S24, S25, S26, S27 in the “supporting information” shows the results of detection of a subtype in the *env* region of CRFs. Table 2 shows the accuracy of subtype detection in the *env* region of CRFs using our method. A comparison of the efficacy of our method of subtype detection in the *env* region with that of the jpHMM method also reveals that in most cases, the performance of our method is comparable to the jpHMM method.

**Table 2:** Accuracy of subtype detection in the *env* region of CRFs.

Subtype in the *env* region of CRFs	Predicted #CRFs having subtype X	Predicted #CRFs using jpHMM	Actual #CRFs containing the subtype	Predicted #sequences containing subtype X	Actual #sequences containing subtype X	Sensitivity
A	16	17	18	376	383	98.17%
B	11	12	16	59	86	68.61%
C	4	4	4	24	24	100.0%
D	3	3	5	9	23	39.13%
F	9	9	10	52	54	96.30%
G	10	13	14	59	69	85.51%
H	2	2	2	8	8	100%
J	2	2	3	20	23	86.96%

.

For subtype A detection, both our method as well as the jpHMM method are unable to detect a small fragment (less than 200 nucleotides) of subtype A2 in CRF21_A2D. This can be attributed to the small number of sequences of subtype A2 available for training as well as due to the short length of the A2 fragment. Our method is also unable to detect subtype A in the *env* region of CRF03_AB even though the fragment is detected by the jpHMM method. CRFs containing crf01_AE were not included in the count of CRFs containing subtype A.

Many of the CRFs have very short fragments of subtype B in the *env* region. Hence we are able to detect this subtype in eleven out of the sixteen CRFs that contain this subtype. In the case of CRF12_BF, we were able to detect the short fragment (179 nucleotides) of subtype B in only two of the seven sequences available in contrast to the jpHMM method which was able to detect the subtype in four sequences. Both our method as well as the jpHMM method was unable to detect very short fragments (less than 105 nucleotides) of subtype B in CRF20_BG,CRF23_BG,CRF24_BG and CRF_44_BF.

Our method was also able to detect subtypes C, F, J, G and H in the *env* region of CRFs with high sensitivity. However, in the first three cases, both our method, as well as the jpHMM method, was unable to detect the corresponding subtype in the *env* region of one CRF because of the very short segment length of the subtype in the CRF. The large drop in sensitivity for subtype D detection can be attributed to the inability of our method (and the jpHMM method) to detect a very short fragment (having a length of 117 nucleotides) of that subtype in all the eleven sequences of CRF35_AD.

In some cases where our subtype detection method in the *env* region failed, the subtype fragment was found to span the end (or beginning) of the *env* region and the beginning of the *nef* (or end of the *vpu*) region. The short length of the subtype in the *env* region was insufficient to contain adequate signatures associated with the subtype that could be detected by our method. However, it is quite likely that an inclusion of the *env* as well as the *nef* (or *vpu*) region would have provided a subtype segment of sufficient length for its associated signature to be detected by our method. In a few cases of subtype detection in CRFs, some CRF01_AE sequences are seen to have Z-scores above the threshold Tn. A large fraction of the *env* region of this CRF consists of an unclassified segment (E) which does not have any full length sequences associated with it. Hence, it is plausible that the training sets for those specific subtypes were unable to appropriately distinguish signatures from this segment from signatures associated with the corresponding subtype. This problem may be resolved by including CRF01_AE sequences in the negative training sets for all subtypes.

## Discussion

We have shown that profile Hidden Markov Models can be successfully employed to classify HIV-1 strains belonging to the group M into different subtypes. Our method does not rely on the choice of an arbitrary threshold. We have also demonstrated that the method is particularly effective in determining the subtype composition of different CRF strains. We used the *gag-pol* and *env* regions for our analysis because those are the regions which contain many of the breakpoints denoting recombination in the CRFs. Accurate classification of a HIV-1 sequence is more challenging when only a fraction of the genome is available. Quite often, only a part of the viral genome from a new strain is sequenced and it therefore becomes necessary to classify it accurately on the basis of the sequenced region. The large size of the *whole* genome makes it impossible to use tools like HMMER3 to classify the sequence. Moreover, since a fraction of the complete genome (either the *gag-pol* or the *env* regions) can be used to classify the sequence much faster and *without* compromising on the classification accuracy, it is far more advantageous to use a region of the genome instead of the whole genome for classification purposes.

Another advantage of this method is that it can be used for subtype classification even if a small number of sequences are available for a particular subtype. The positive profile HMM can be effectively constructed from as few as six sequences as long as the sequences contain sufficient diversity for the positive (and negative) profiles to capture the unique features associated with the subtype. Even in the case of subtypes H and J which contain very few sequences, we find that a pHMM built (using the *gag-pol* region) with 3 out of the 4 sequences of H (or 2 out of the 4 sequences of J) can accurately classify the remaining sequences as is evident from Figure S5 in “supporting information”. Similarly, for the *env* region, a profile made with 3 out of 4 sequences of H (or J) can classify the remaining sequence accurately as is seen in Figures S18, S19 in the “supporting information”.

The sequences labelled as “unclassified” in the database were not found to be associated with any subtype in the sense that their Z-scores were always negative. However, some unclassified sequences show strong similarity with certain subtypes as manifest through their Z-scores which were very close to zero. For instance, the unclassified strain AY046058 has a Z-score which is very close to the zero threshold when the positive profile is constructed for subtype A. This suggests that this strain share similarities with the A-subtype and may explain why it clusters with the A-subtype in phylogenetic studies (Pandit *et al.,* 2010). Another unclassified sequence (accession number FJ388921) share similarities with sequences belonging to the F subtype, as evident from their Z-scores (see Fig.S3 in “supporting information”). This is also consistent with results from previous analysis where these sequences have been found to cluster with subtype K and form the sister group of subtype F.

The performance of our pHMM based classification method is also better than the subtype analyser method based on construction of PSSMs [16], [17]. Their method, which is used to assign sequence subtypes to the PR and RT sequences belonging to the *Pol* region, depends on the choice of a scoring threshold as well as a choice of positive and negative discriminant thresholds. Even for optimal choice of such thresholds, the accuracy of classification of pure subtypes was 98.6%. However, this method was far less accurate (60%) in identifying CRFs. The STAR-rec method which allowed for improved accuracy in identification of CRFs had an accuracy of about 93–95% for sub-typing of both pure subtypes and CRFs, but the accuracy of their CRF subtype detection method was obtained on the basis of only 20 CRF sequences. The STAR program is also restricted to detect subtypes in only the Pol region of CRFs. Moreover, the program cannot correctly determine the subtype composition for many of the CRF strains currently available in the HIV database, and predicts them to belong to pure subtypes. In comparison, our improved pHMM based method can classify pure sequences with 100% accuracy based on the sign of the Z-score of the query sequence, since the Z-score threshold is always fixed at zero. Moreover, our method can be adopted without modification to accurately identify the subtype composition of most CRFs with the accuracy of subtype detection in CRFs being above 95% for most subtypes. Determination of the subtype composition of CRFs may fail only in those rare circumstances when the subtype present has a very short segment in the region being analysed or if that region is constituted entirely of one subtype only and the other subtype(s) are present in a different region of the CRF. The problem can be easily resolved by constructing pHMMs from other regions of the genome and repeating the analysis. By combining the results of such analysis, it is straightforward to infer the subtype composition of the CRF accurately.

Although the jpHMM method [26], [27] is primarily geared for identifying breakpoints in CRFs, it can also be used for detecting subtypes. An extensive comparison of the accuracy of that classification method with ours is not possible since those papers do not provide sufficient data on subtype classification for all real HIV-1 strains. While being based on pHMM, our method of classification differs from the jpHMM method of Schultz et al. In our case, 100% classification accuracy is achieved by introducing a negative training set in addition to the positive training set used in the standard pHMM method. As far as subtype detection in CRFs is concerned, our pHMM method performs as well as the jpHMM method. Moreover, in our method, once the positive and negative profiles are created, it is easy to generate the Z-score (on the basis of which the query sequence is classified) for an entire database of test sequences simultaneously. This makes it possible to classify batches of query sequences rather than do so one query sequenceat a time.

**Figure 8:**
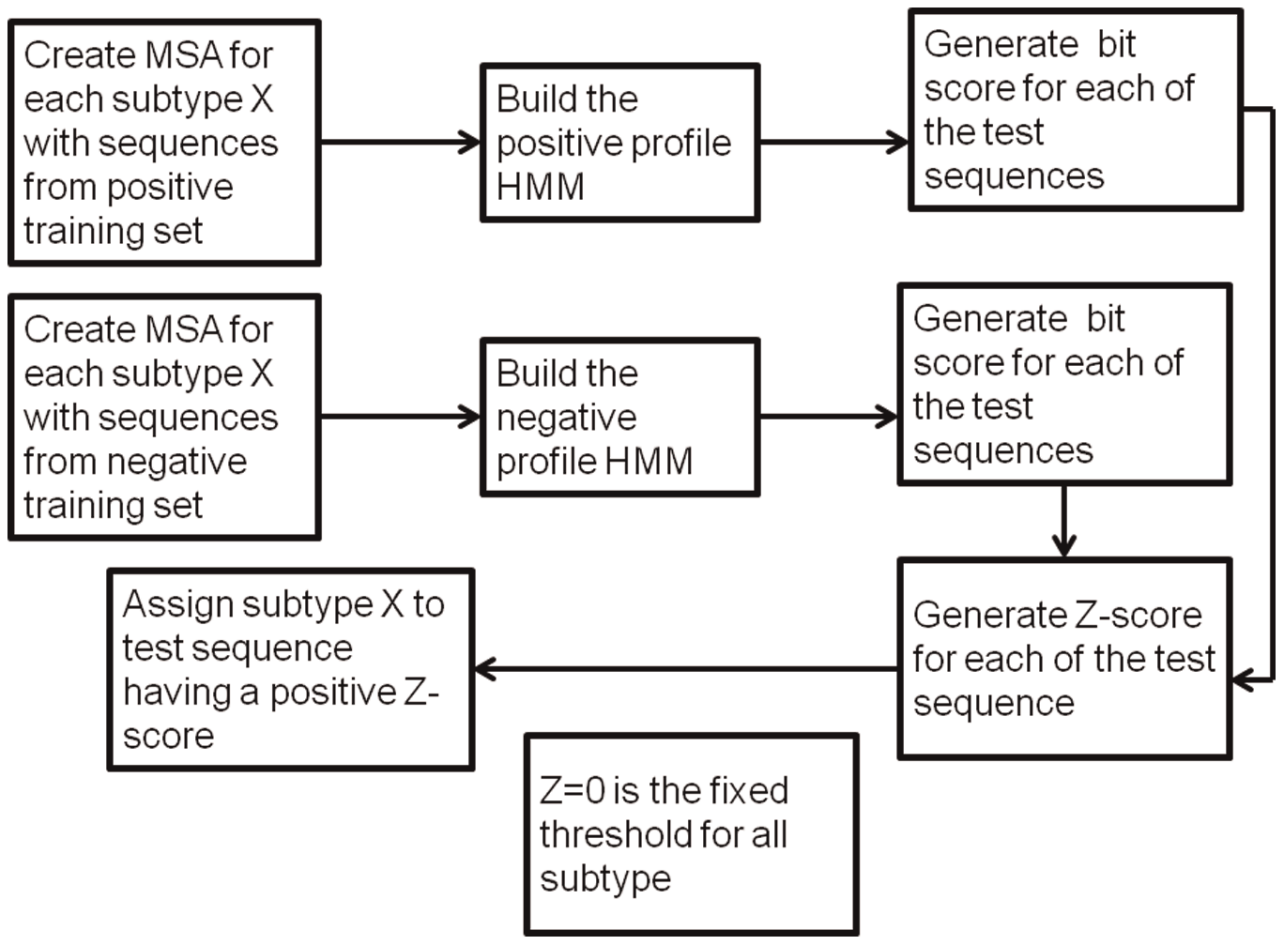
Flowchart showing the improved method of classification using pHMMs.

The pHMM based-method described in this paper provides a unified and efficient method of determining the subtypes of unknown HIV-1 sequences as well as CRFs. We believe it will prove useful to HIV researchers for understanding the distribution pattern of different types of HIV and for developing more effective and targeted therapies against the disease.

## Materials and Methods

All available HIV-1 genome sequences including CRF sequences were downloaded from the Los Alamos HIV database. For each subtype, six sequences were selected at random to build the training set. Multiple sequence alignments (MSA) of the *gag-pol* segment of these nucleotide sequences were created using the MUSCLE 3.7 package [36]. A pHMM was then generated for each of the MSA using the *hmmbuild* program from the HMMER3 package [37]. The log-odd or bit score for each of the 1511 genome sequences in the database (six of which were used for the training set and the rest made up the test set, for each subtype) was generated by the *hmmsearch* program in HMMER3. For pure (i.e. non-CRF) sequences, we developed a classification method based on the bit score. In this method, all sequences have a positive bit score and the membership of a sequence in the corresponding subtype was dependent on its bit score and a threshold score which distinguished the true positives from the true negatives. If the bit-score was found to be greater than the threshold score, the corresponding query sequence was predicted to belong to the subtype for which the pHMM was constructed. This threshold was chosen arbitrarily to lie between the scores of the high-scoring and low-scoring clusters of query sequences. Overlap between these two clusters signifies imperfect classification. Even though this threshold varied for classification of different subtypes, we were able to unambiguously assign a threshold score for all subtypes (except B and D) which allowed for accurate discrimination between true positives and true negatives.

The standard method of classification described above was unable to discriminate between sequences belonging to subtypes B and D with 100% accuracy. We therefore used an improved method for subtype classification that made use of a positive as well as a negative profile HMM. Creation of these two different profiles allowed us to capture the unique signatures associated with a specific subtype and therefore more effectively distinguish subtypes such as B and D which show a higher level of sequence similarity than is observed between any other subtypes. For subtypes other than B and D, a positive training set was constructed for a specific subtype using six randomly chosen sequences (avoiding redundancy) known to belong to that subtype. A positive profile HMM was created for that subtype (say subtype X) from the MSA of the positive training set, using the *hmmbuild* program in HMMER3. A negative training set was also constructed using two sequences of the *gag-pol* segment from each of the other subtypes. A negative profile HMM was then constructed for subtype X from the MSA of the negative training set. For classification of a query sequence using this method, a Z-score is obtained by subtracting the bit score of the query sequence generated by the negative profile HMM from the bit score of the query sequence generated by the corresponding positive profile HMM. This Z score is a measure of subtype specific signal and is an effective measure for accurately classifying the query sequence. This method also has the advantage of fixing the threshold score (for all subtype classification) to zero. A query sequence is then predicted to belong to subtype X if its Z-score is positive when the positive pHMM is constructed using sequences belonging to subtype X. Fig.8 gives the flowchart (see also File S1 in the “Supporting information”) of the improved classification method. Even with this method, some sequences belonging to subtype D were misclassified as belonging to subtype B. However, this problem was avoided when the number of sequences belonging to subtype B in the negative training set (weight of B in the negative training set) was increased from two to six. Subtypes such as A and F are further divided into sub-subtypes. In the case of A, the positive training set was created using two randomly chosen sequences from A (i.e. those not belonging to either A1 or A2) and two randomly chosen sequences from each of the sub-subtypes A1 and A2. In case of F, the positive training set was created using six randomly chosen sequences from F1 sub-subtype only. Table S1,S2, S3, S4 in “Supporting information” gives the numbers of sequences used to create the positive and negative training sets for each subtype. The pHMM profiles for the positive and negative training sets for each subtype, constructed using the *gag-pol* region, can be obtained from http://ccbb.jnu.ac.in/hiv1/pure-phmms.The pHMM profiles for the positive and negative training sets for each subtype, constructed using the *env* region, can be obtained from http://ccbb.jnu.ac.in/hiv1/pure-phmms-env.

We used the improved method described above to determine the subtype composition of CRFs. In some cases, the number of sequences used to build the positive and negative profiles had to be increased to enhance the sensitivity required for capturing unique signatures associated with a subtype from a small subtype fragment (see Tables S5, S6 and Tables S13, S14 in “Supporting information”). For the purpose of determining whether a given subtype (X say) was represented in the *gag-pol* (or *env*) region of a CRF, we defined two thresholds: (i) Tp which is the minimum Z score of all the pure sequences belonging to X and (ii) Tn which is maximum Z score of all the pure sequences (excluding unclassified sequences) that do not belong to the X subtype. For determination of Tn, we ignored the unclassified (U) sequences. If the query CRF strain returns a Z score greater than Tn and less than Tp, we predict that some portion of the *gag-pol* (or *env*) segment of the X subtype is present in the CRF strain. If the CRF strains returns a Z score greater than Tp then the *gag-pol* (or *env*) region of this strain can be predicted to consist, either entirely of the X subtype, or of a mixture in which the major fraction of the *gag-pol* (or env) region is of X subtype. Table S12 and Table S15 give the thresholds for detection of subtypes in the *gag-pol* and *env* region of CRFs respectively. The method was tested on all 2033 CRF sequences available in the database to determine the subtype composition of a CRF in the *gag-pol* and *env* regions. The method can be repeated using positive training sets made up of different subtypes to determine the subtype composition of the *gag-pol* and *env* regions of the CRF. The subtype composition of other segments of the CRF can also be determined in a similar manner. This method can fail to detect a particular subtype in a CRF only if the length of the segment of that subtype is too small to contain subtype specific signatures that have been captured by the pHMM. In determining the subtype composition of CRFs, we did not distinguish between sub-subtypes A1 and A2 of subtype A or F1 and F2 of subtype F. In the former case, the positive training set was constructed using 2 sequences of subtype A and 2 sequences each of sub-subtypes A1 and A2. In the latter case, the positive training set was constructed using 8 sequences from sub-subtype F1 and 2 from sub-subtype F2. The pHMM profiles for the positive and negative training sets, constructed using the *gag-pol* and *env* regions, and used for identifying the subtype composition of CRFs, can be obtained from http://ccbb.jnu.ac.in/hiv1/crf-phmms and http://ccbb.jnu.ac.in/hiv1/env-crf-phmms respectively.

## Supporting Information

Figure S1 **Subtype A classification using the improved method.** (a) Distribution of Z-scores of group M sequences. (b) Frequency distribution of Z-scores when six sequences from subtype A are used to build the positive training set.
(PDF)

Figure S2 **Subtype C classification using the improved method.** (a) Distribution of Z-scores of group M sequences. (b) Frequency distribution of Z-scores when six sequences from subtype C are used to build the positive training set.
(PDF)

Figure S3 **Sub-subtype F1 classification using the improved method.** (a) Distribution of Z-scores of group M sequences. (b) Frequency distribution of Z-scores when six sequences from subtype F1 are used to build the positive training set. The four sequences of subtype F2 have a Z-score close to zero indicating their close similarity to sequences of subtype F1.
(PDF)

Figure S4 **Subtype G classification using the improved method.** (a) Distribution of Z-scores of group M sequences. (b) Frequency distribution of Z-scores when six sequences from subtype G are used to build the positive training set.
(PDF)

Figure S5 **Classification of subtypes (a) H (b) J using the improved method.** Three out of four sequences are used to construct the positive profile HMM for H and two out of four sequences are used to construct the positive profile HMM for J. In both cases, the remaining sequences are correctly classified.
(PDF)

Figure S6 **Detection of subtype A in the** ***gag-pol*** **region of CRF’s**. The figures show the distribution of Z-scores of (a) pure sequences and (b)-(e): all CRF strains when the positive training set is constructed using two sequences each belonging to A,A1 and A2. Abbreviations are used to label CRF strains in the X-axis. For example, the label 02 refers to CRF02_AG and 18 refer to CRF18_cpx etc. The solid and dashed lines correspond to the thresholds Tp and Tn respectively.
(PDF)

Figure S7 **Detection of subtype C in the** ***gag-pol*** **region of CRF’s**. The figures show the distribution of Z-scores of (a) pure sequences and (b)-(e) all CRF strains when the positive training set is constructed using sixteen sequences belonging to C. The solid and dashed lines correspond to the thresholds Tp and Tn respectively.
(PDF)

Figure S8 **Detection of subtype D in the** ***gag-pol*** **region of CRF’s**. The figures show the distribution of Z-scores of (a) pure sequences and (b)-(e) all CRF strains when the positive training set is constructed using fourteen sequences belonging to D. The solid and dashed lines correspond to the thresholds Tp and Tn respectively. Subtype D is not detected only in CRF16_A2D since very small fragments of D are present in the *gag-pol* region. There are no data points corresponding to CRF41_CD since sequences belonging to that strain are not yet publicly available.
(PDF)

Figure S9 **Detection of subtype G in the** ***gag-pol*** **region of CRF’s**. The figures show the distribution of Z-scores of (a) pure sequences and (b)-(e) all CRF strains when the positive training set is constructed using six sequences belonging to G. The solid and dashed lines correspond to the thresholds Tp and Tn respectively.
(PDF)

Figure S10 **Detection of subtype H in the** ***gag-pol*** **region of CRF’s**. The figures show the distribution of Z-scores of (a) pure sequences and (b)-(e) all CRF strains when the positive training set is constructed using four sequences belonging to H. The solid and dashed lines correspond to the thresholds Tp and Tn respectively.
(PDF)

Figure S11 **Detection of subtype J in the** ***gag-pol*** **region of CRF’s**. The figures show the distribution of Z-scores of (a) pure sequences and (b)-(e) all CRF strains when the positive training set is constructed using three sequences belonging to J. The solid and dashed lines correspond to the thresholds Tp and Tn respectively.
(PDF)

Figure S12 **Subtype A classification using the improved method when the** ***env*** **region is used to construct the positive and negative pHMMs.** Distribution of Z-scores for group M sequences.
(TIF)

Figure S13 **Subtype B classification using the improved method when the** ***env*** **region is used to construct the positive and negative pHMMs.** Distribution of Z-scores for group M sequences.
(TIF)

Figure S14 **Subtype C classification using the improved method when the** ***env*** **region is used to construct the positive and negative pHMMs.** Distribution of Z-scores for group M sequences.
(TIF)

Figure S15 **Subtype D classification using the improved method when the** ***env*** **region is used to construct the positive and negative pHMMs.** Distribution of Z-scores for group M sequences.
(TIF)

Figure S16 **Subtype F1 classification using the improved method when the** ***env*** **region is used to construct the positive and negative pHMMs.** Distribution of Z-scores for group M sequences.
(TIF)

Figure S17 **Subtype G classification using the improved method when the** ***env*** **region is used to construct the positive and negative pHMMs.** Distribution of Z-scores for group M sequences.
(TIF)

Figure S18 **Subtype H classification using the improved method when the** ***env*** **region is used to construct the positive and negative pHMMs.** Distribution of Z-scores for group M sequences.
(TIF)

Figure S19 **Subtype J classification using the improved method when the** ***env*** **region is used to construct the positive and negative pHMMs.** Distribution of Z-scores for group M sequences.
(TIF)

Figure S20 **Detection of subtype A in the** ***env*** **region of CRF’s**. The figures show the distribution of Z-scores of (a) pure sequences and (b)-(e): all CRF strains when the positive training set is constructed using a total of twelve sequences belonging to A,A1 and A2. The solid and dashed lines correspond to the thresholds Tp and Tn respectively.
(PDF)

Figure S21 **Detection of subtype B in the** ***env*** **region of CRF’s**. The figures show the distribution of Z-scores of (a) pure sequences and (b)-(e): all CRF strains when the positive training set is constructed using thirty sequences each belonging to B. The solid and dashed lines correspond to the thresholds Tp and Tn respectively.
(PDF)

Figure S22 **Detection of subtype C in the** ***env*** **region of CRF’s**. The figures show the distribution of Z-scores of (a) pure sequences and (b)-(e) all CRF strains when the positive training set is constructed using ten sequences belonging to C. The solid and dashed lines correspond to the thresholds Tp and Tn respectively.
(PDF)

Figure S23 **Detection of subtype D in the** ***env*** **region of CRF’s**. The figures show the distribution of Z-scores of (a) pure sequences and (b)-(e) all CRF strains when the positive training set is constructed using twenty sequences belonging to D. The solid and dashed lines correspond to the thresholds Tp and Tn respectively.
(PDF)

Figure S24 **Detection of subtype F in the** ***env*** **region of CRF’s**. The figures show the distribution of Z-scores of (a) pure sequences and (b)-(e) all CRF strains when the positive training set is constructed using twelve sequences belonging to F. The solid and dashed lines correspond to the thresholds Tp and Tn respectively.
(PDF)

Figure S25 **Detection of subtype G in the** ***env*** **region of CRF’s**. The figures show the distribution of Z-scores of (a) pure sequences and (b)-(e) all CRF strains when the positive training set is constructed using twelve sequences belonging to G. The solid and dashed lines correspond to the thresholds Tp and Tn respectively.
(PDF)

Figure S26 **Detection of subtype H in the** ***env*** **region of CRF’s**. The figures show the distribution of Z-scores of (a) pure sequences and (b)-(e) all CRF strains when the positive training set is constructed using four sequences belonging to H. The solid and dashed lines correspond to the thresholds Tp and Tn respectively.
(PDF)

Figure S27 **Detection of subtype J in the** ***env*** **region of CRF’s**. The figures show the distribution of Z-scores of (a) pure sequences and (b)-(e) all CRF strains when the positive training set is constructed using four sequences belonging to J. The solid and dashed lines correspond to the thresholds Tp and Tn respectively.
(PDF)

Table S1 **Accession numbers of sequences making up the positive training set for all sub-types except H & J when the** ***gag-pol*** **region is used for classification.**
(PDF)

Table S2 **Accession numbers of sequences making up the positive training set for sub-type H and J when the** ***gag-pol*** **region is used for classification.** Since there are only 4 sequences of the *gag-pol* region for H and 3 for J, 3 of the 4 sequences of H and 2 of the 3 sequences of J were used to build the respective pHMMs which were then successful in classifying the remaining sequences.
(PDF)

Table S3 **Accession numbers of sequences making up the negative training set for all sub-types except D when the** ***gag-pol*** **region is used for classification.**
(PDF)

Table S4 **Accession numbers of sequences making up the negative training set for sub-type D when the** ***gag-pol*** **region is used for classification.**
(PDF)

Table S5 **Number of sequences making up the positive training set used for determining whether a given sub-type (X) is present in the** ***gag-pol*** **region of a CRF.**
(PDF)

Table S6 **Number of sequences of each sub-type making up the negative training set used for determining whether a given sub-type (X) is present in the** ***gag-pol*** **region of a CRF.**
(PDF)

Table S7 **Accession numbers of sequences making up the positive training set for all sub-types, except H and J, when the** ***env*** **region is used for classification.**
(PDF)

Table S8 **Accession numbers of sequences making up the negative training set for all sub-types, except H and J, when the** ***env*** **region is used for classification.**
(PDF)

Table S9 **Accession numbers of sequences making up the positive training set for sub-type H and J when the** ***env*** **region is used for classification.** Since there are only 4 sequences of the *env* region for both H and J, three were used to build the pHMM which was then successful in classifying the remaining sequence. Note that a choice of ***any*** 3 of the 4 *env* sequences can be used to build a pHMM which can successfully classify the remaining one.
(PDF)

Table S10 **Accession numbers of sequences making up the negative training set for sub-type H when the** ***env*** **region is used for classification.**
(PDF)

Table S11 **Accession numbers of sequences making up the negative training set for sub-type J when the** ***env*** **region is used for classification.**
(PDF)

Table S12 **Thresholds for detection of sub-types in the** ***gag-pol*** **region of CRF strains.**
(PDF)

Table S13 **Number of sequences making up the positive training set used for determining whether a given sub-type (X) is present in the** ***env*** **region of a CRF.**
(PDF)

Table S14 **Number of sequences of each sub-type making up the negative training set used for determining whether a given sub-type (X) is present in the** ***env*** **region of a CRF.**
(PDF)

Table S15 **Thresholds for detection of sub-types in the** ***env*** **region of CRF strains.**
(PDF)

File S1 **Instructions for classifying a query sequence (Q) using our pHMM method.**
(PDF)
