# Supplementary figures and images for: Classification of HIV-1 Sequences Using Profile Hidden Markov Models
Source: PLoS One. 2012 May 18;7(5):e36566. doi: 10.1371/journal.pone.0036566 (PMC3356369; doi:10.1371/journal.pone.0036566)

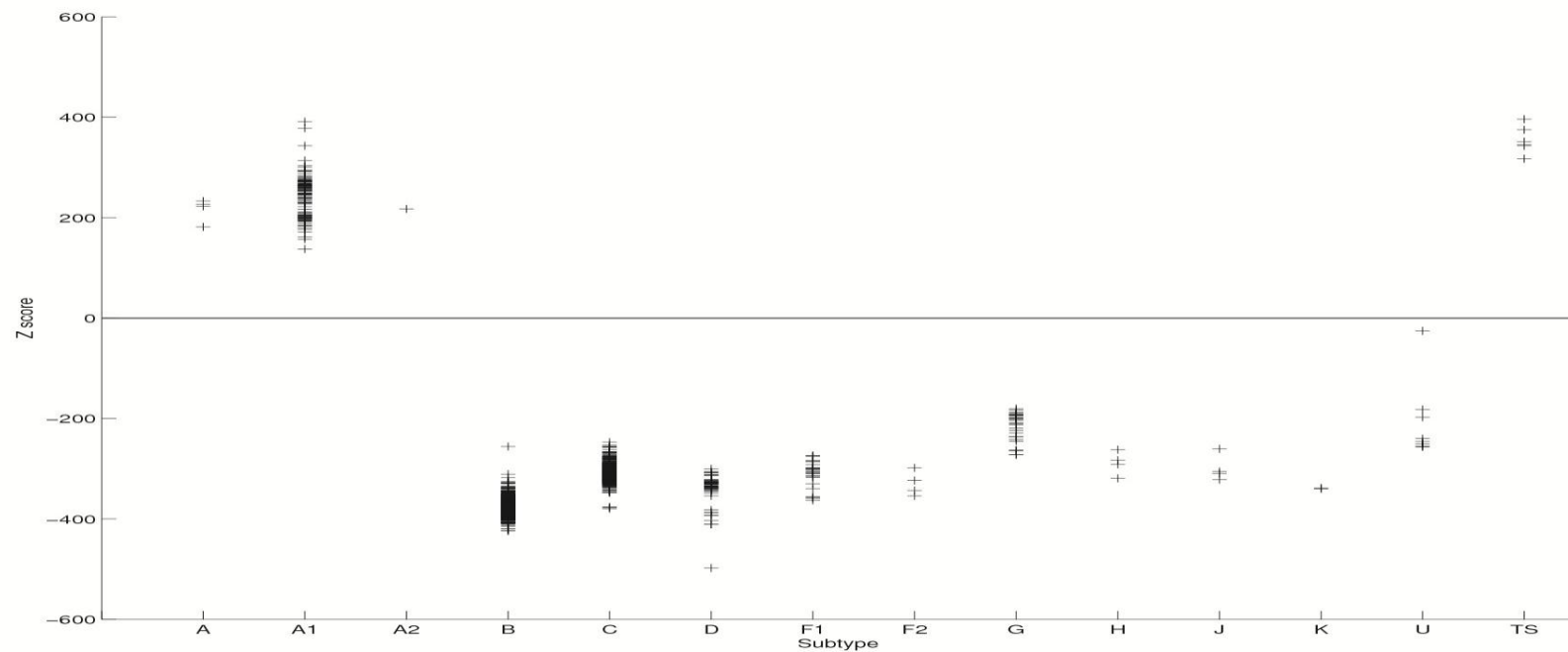

(a)

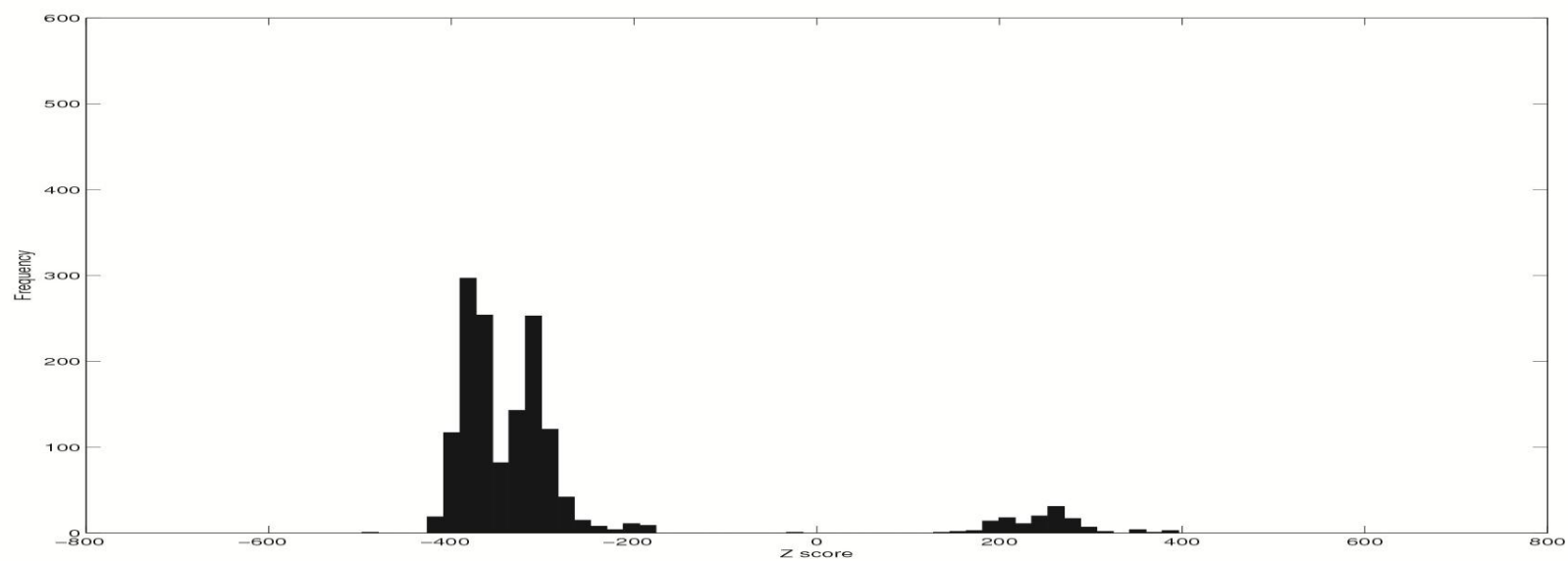

(b)

Supplement: Figure S1 — Subtype A classification using the improved method. (a) Distribution of Z-scores of group M sequences. (b) Frequency distribution of Z-scores when six sequences from subtype A are used to build the positive training set. (PDF) [file pone.0036566.s001.pdf]

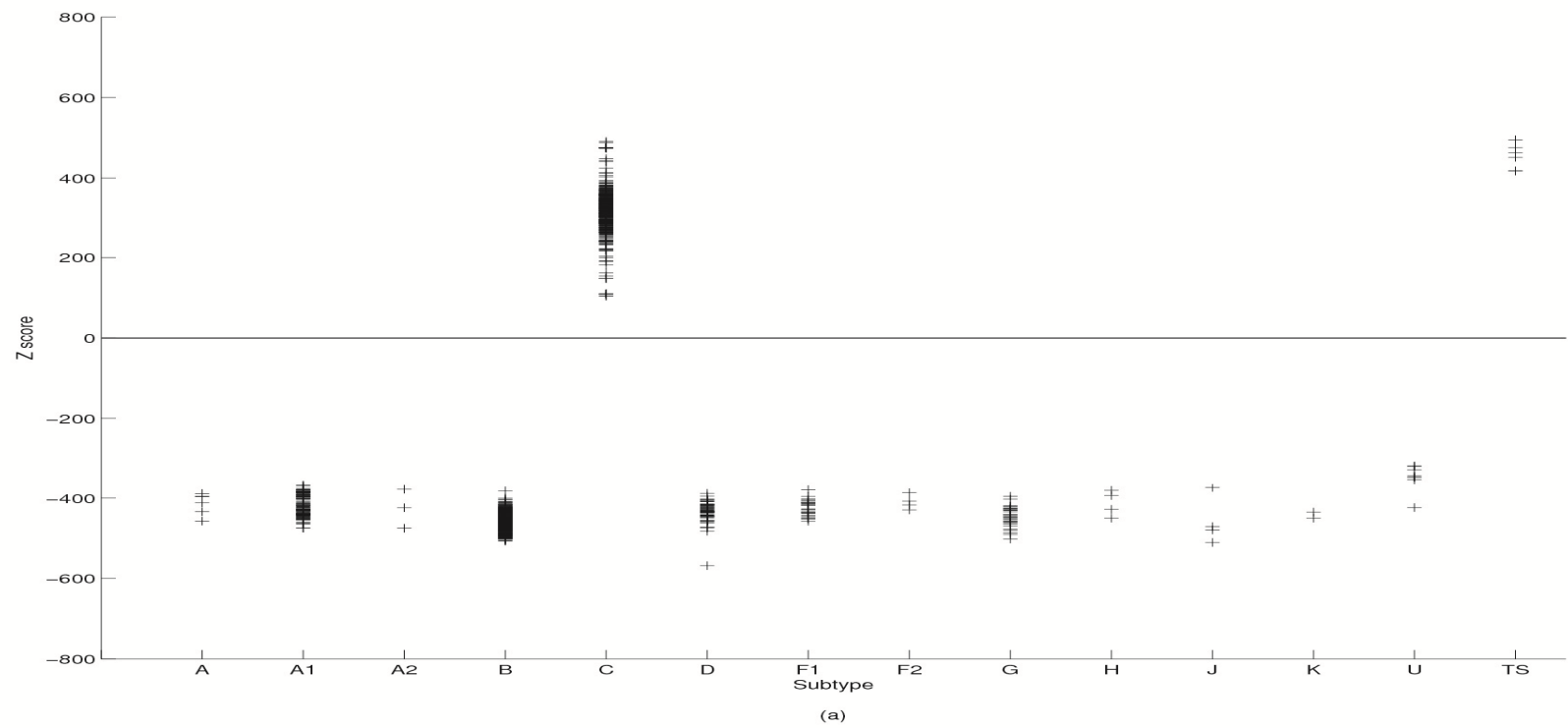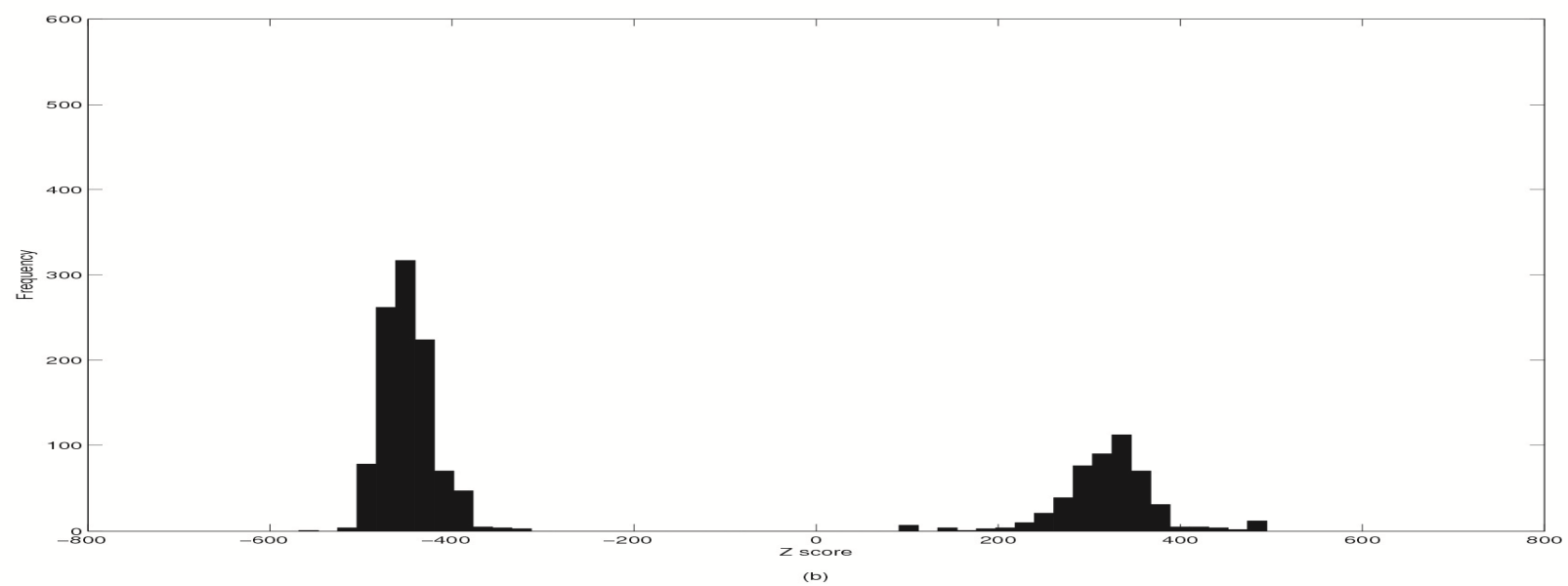

Supplement: Figure S2 — Subtype C classification using the improved method. (a) Distribution of Z-scores of group M sequences. (b) Frequency distribution of Z-scores when six sequences from subtype C are used to build the positive training set. (PDF) [file pone.0036566.s002.pdf]

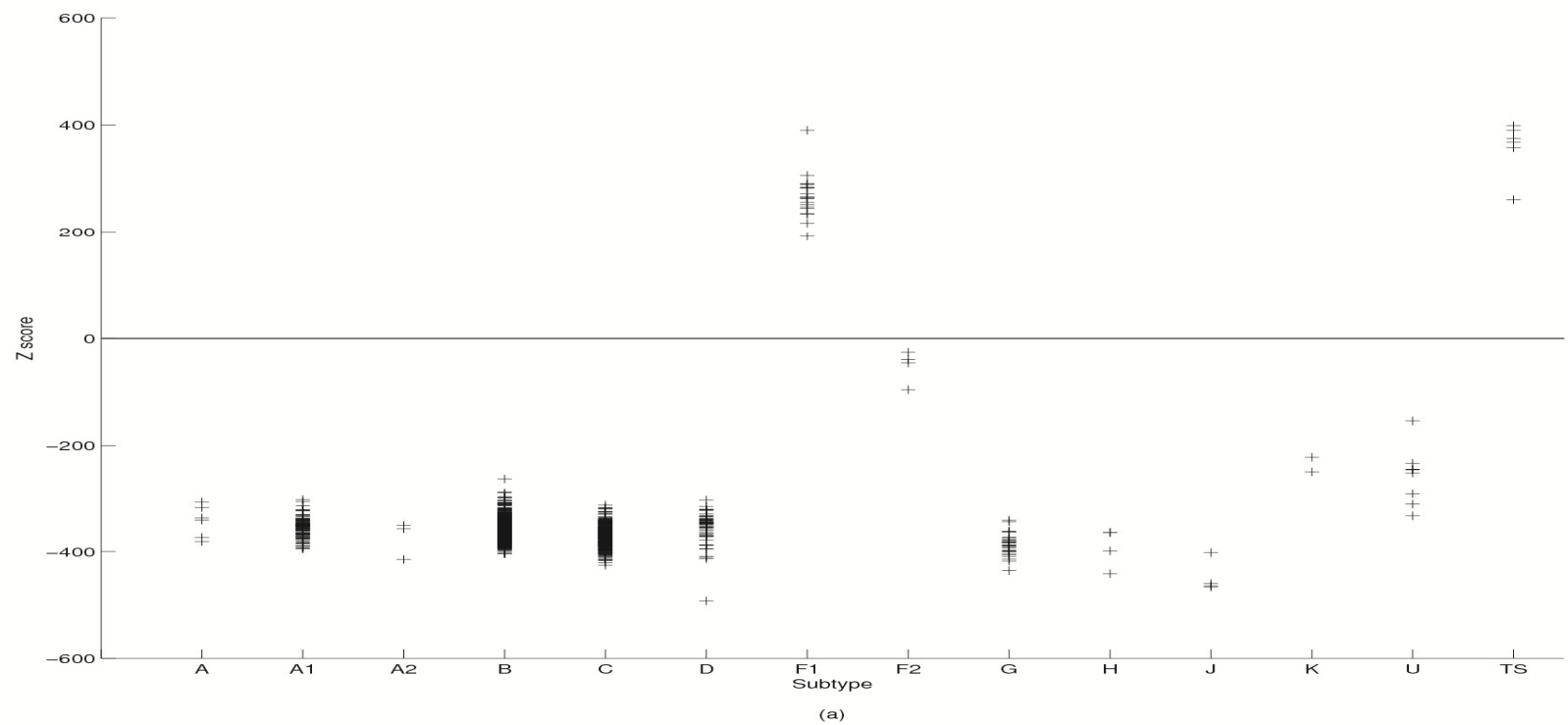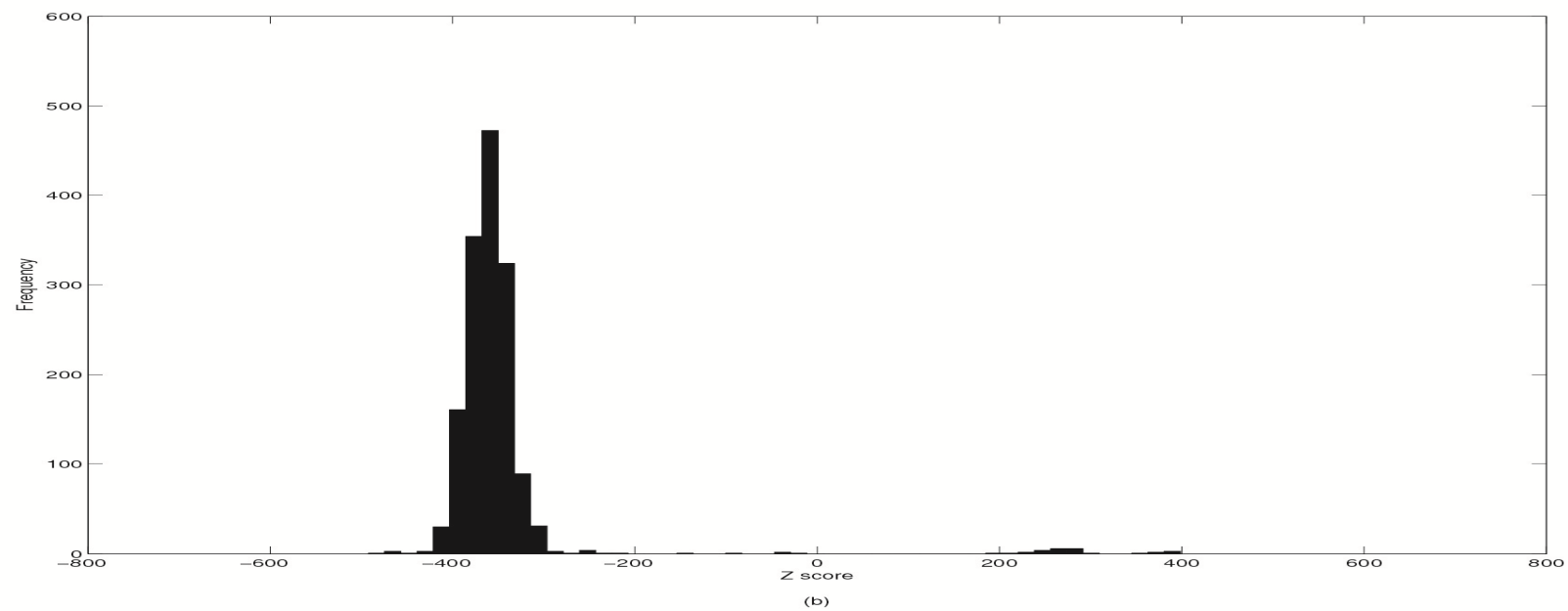

Supplement: Figure S3 — Sub-subtype F1 classification using the improved method. (a) Distribution of Z-scores of group M sequences. (b) Frequency distribution of Z-scores when six sequences from subtype F1 are used to build the positive training set. The four sequences of subtype F2 have a Z-score close to zero indicating their close similarity to sequences of subtype F1. (PDF) [file pone.0036566.s003.pdf]

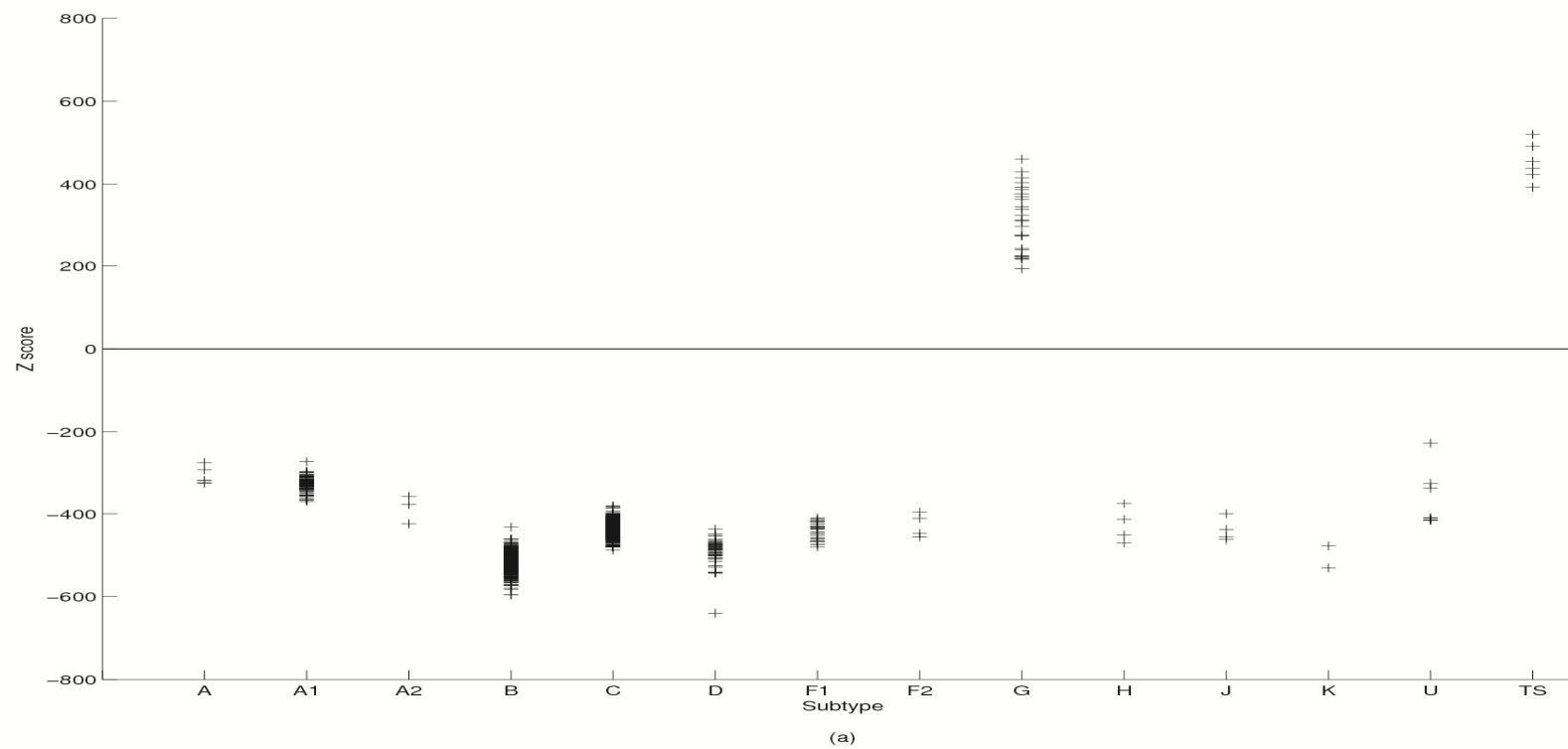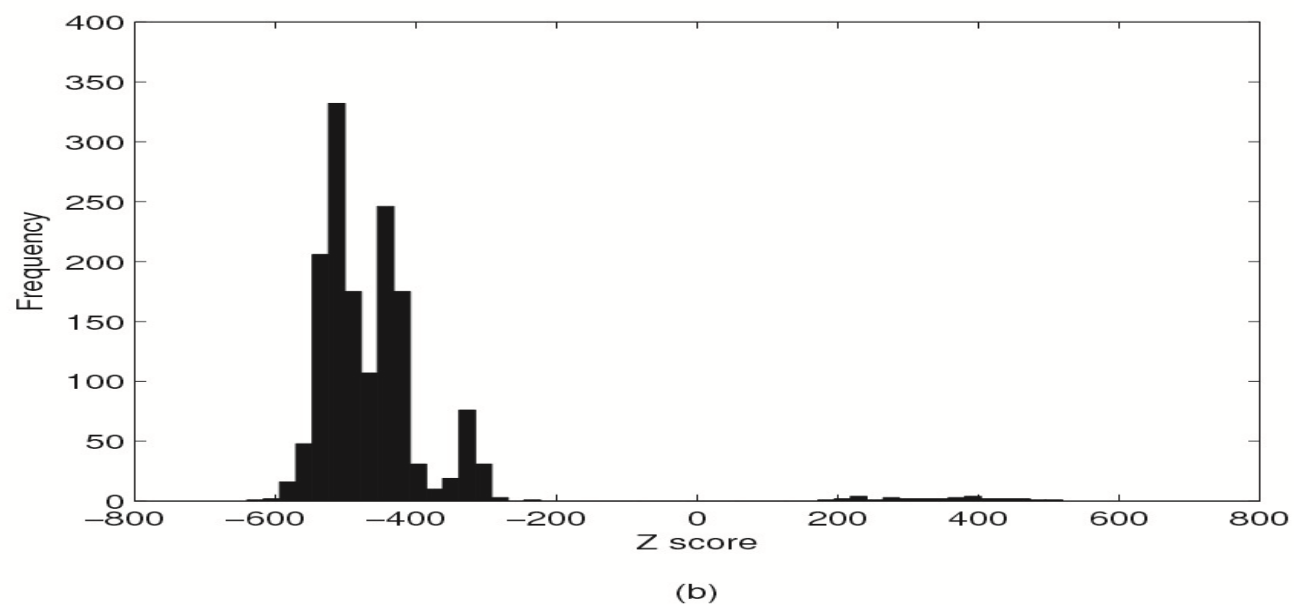

Supplement: Figure S4 — Subtype G classification using the improved method. (a) Distribution of Z-scores of group M sequences. (b) Frequency distribution of Z-scores when six sequences from subtype G are used to build the positive training set. (PDF) [file pone.0036566.s004.pdf]

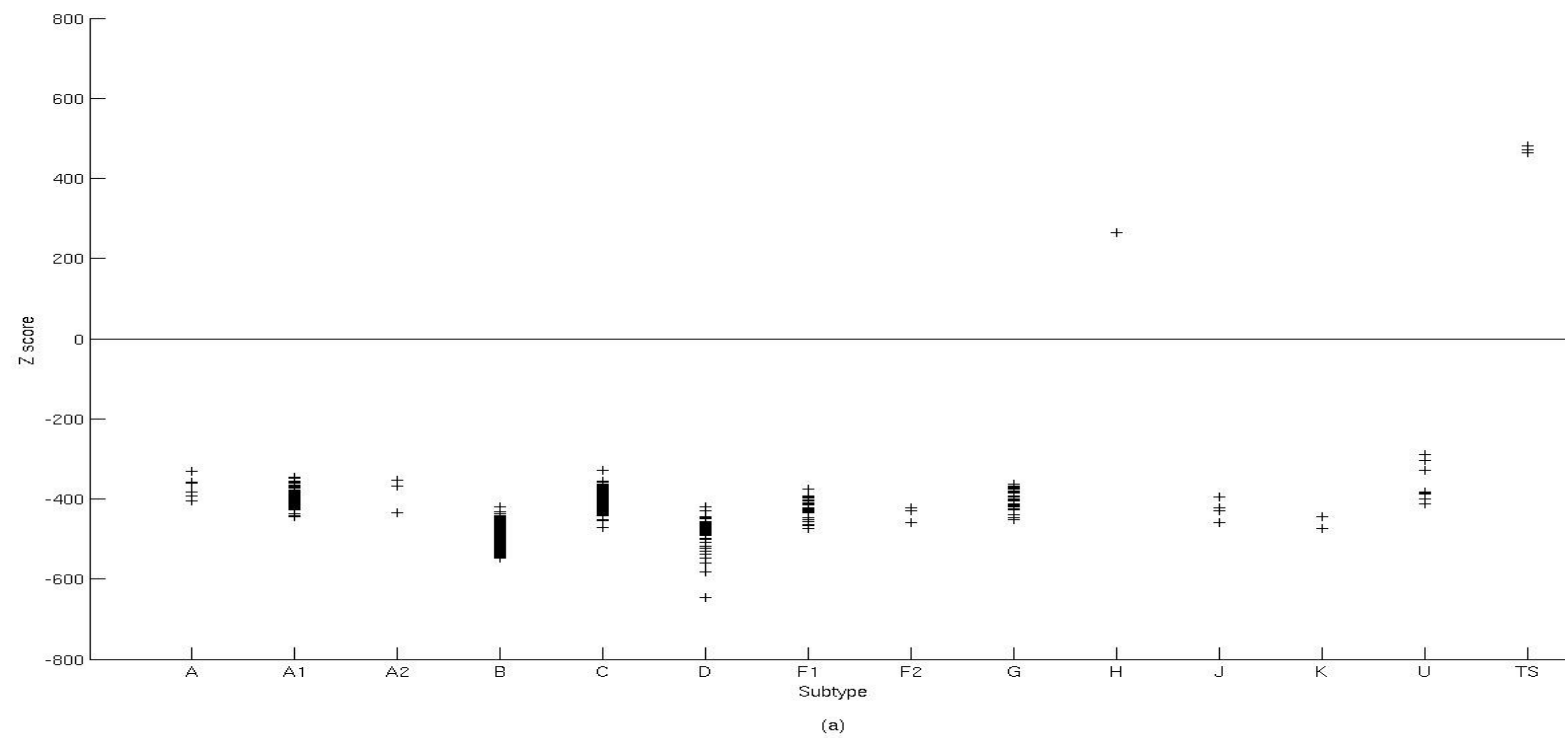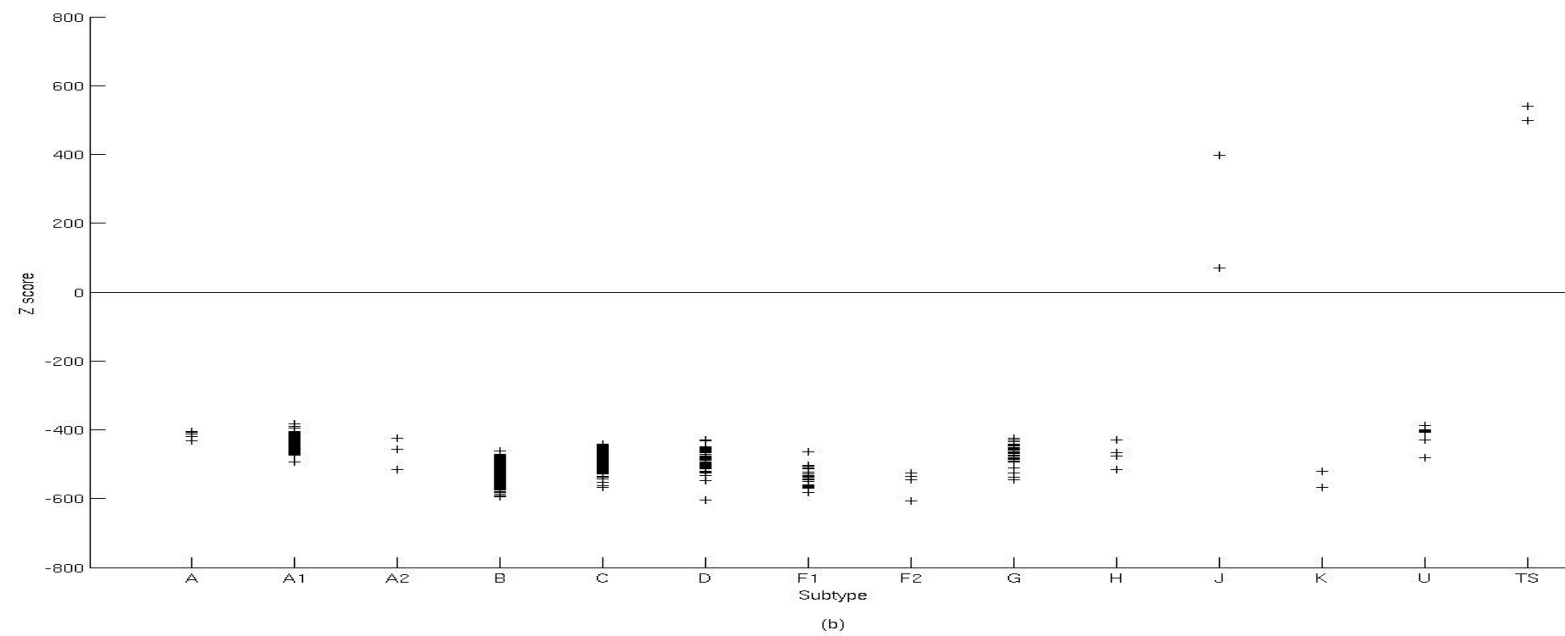

Supplement: Figure S5 — Classification of subtypes (a) H (b) J using the improved method. Three out of four sequences are used to construct the positive profile HMM for H and two out of four sequences are used to construct the positive profile HMM for J. In both cases, the remaining sequences are correctly classified. (PDF) [file pone.0036566.s005.pdf]

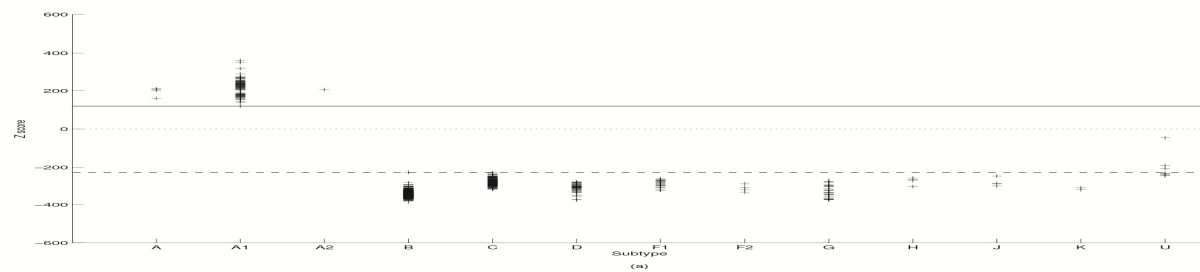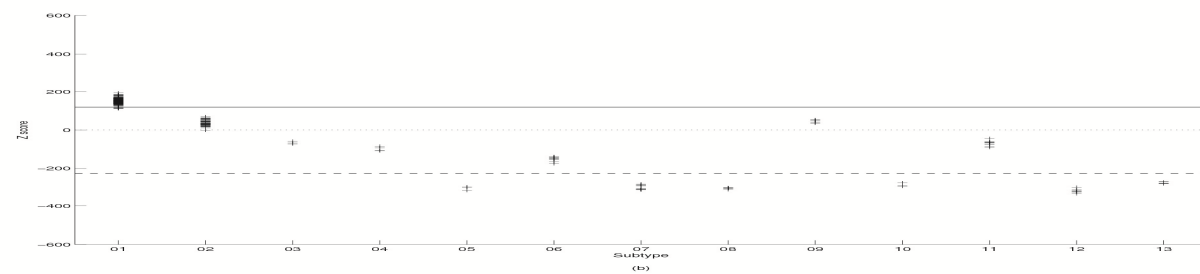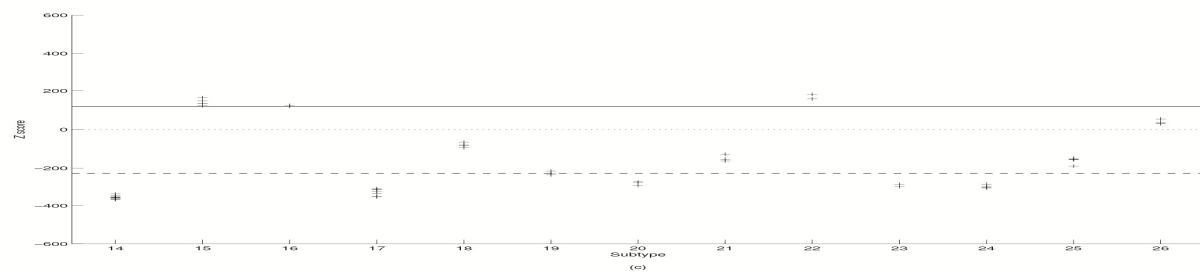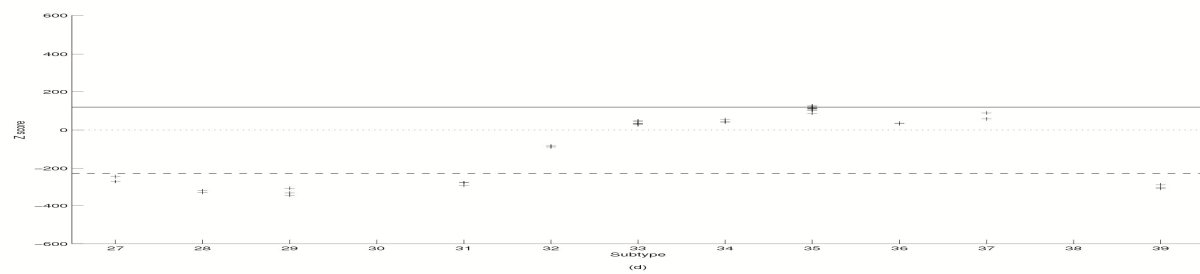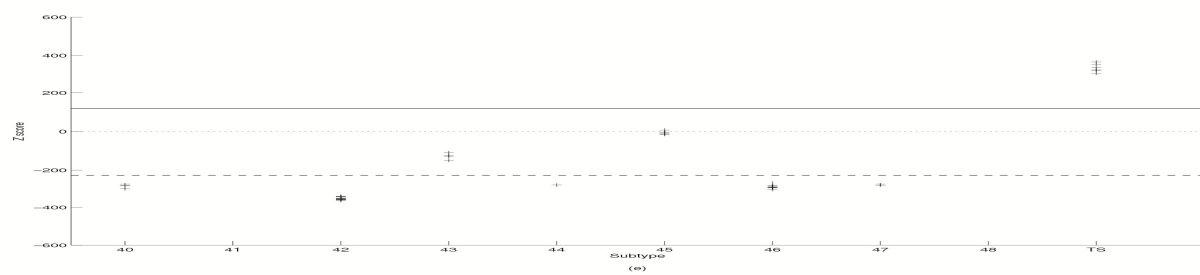

Supplement: Figure S6 — Detection of subtype A in the gag-pol region of CRF’s. The figures show the distribution of Z-scores of (a) pure sequences and (b)-(e): all CRF strains when the positive training set is constructed using two sequences each belonging to A,A1 and A2. Abbreviations are used to label CRF strains in the X-axis. For example, the label 02 refers to CRF02_AG and 18 refer to CRF18_cpx etc. The solid and dashed lines correspond to the thresholds Tp and Tn respectively. (PDF) [file pone.0036566.s006.pdf]

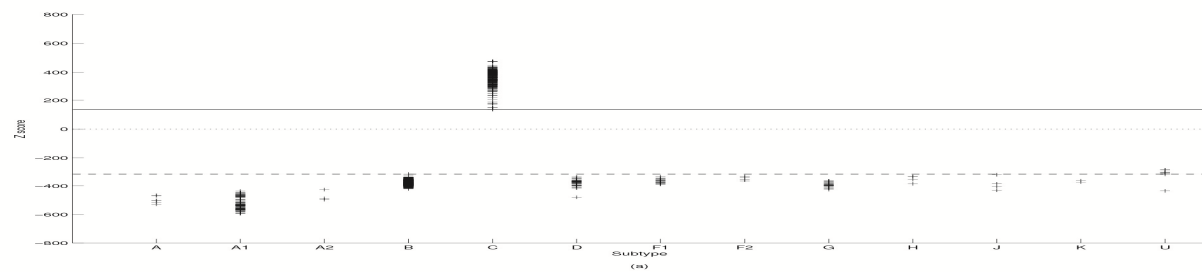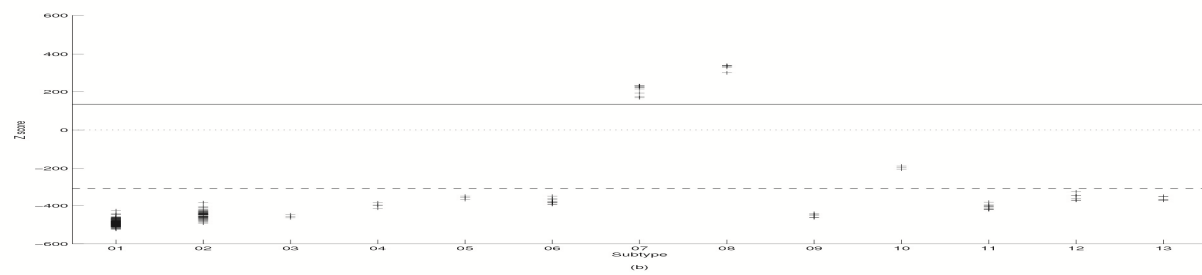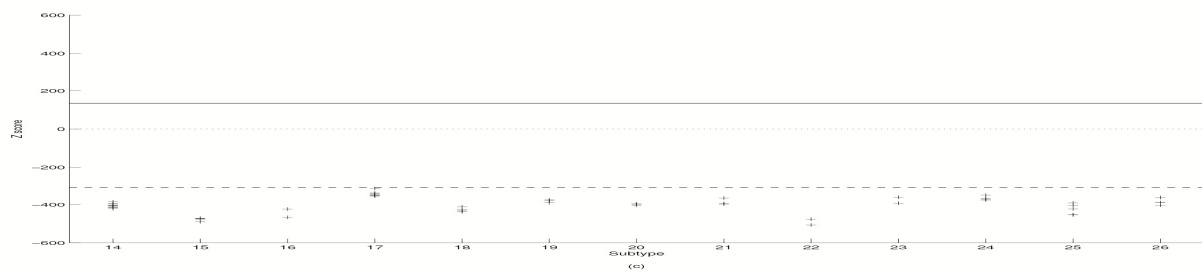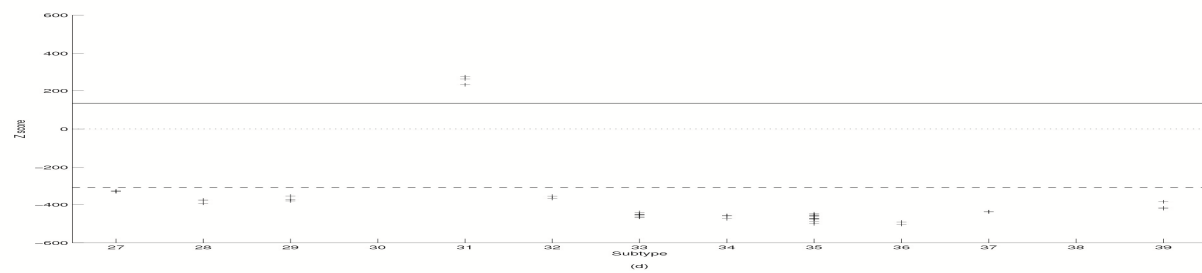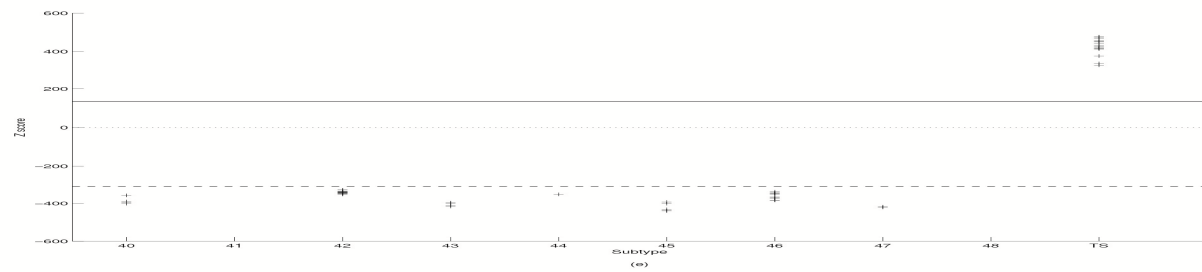

Supplement: Figure S7 — Detection of subtype C in the gag-pol region of CRF’s. The figures show the distribution of Z-scores of (a) pure sequences and (b)-(e) all CRF strains when the positive training set is constructed using sixteen sequences belonging to C. The solid and dashed lines correspond to the thresholds Tp and Tn respectively. (PDF) [file pone.0036566.s007.pdf]

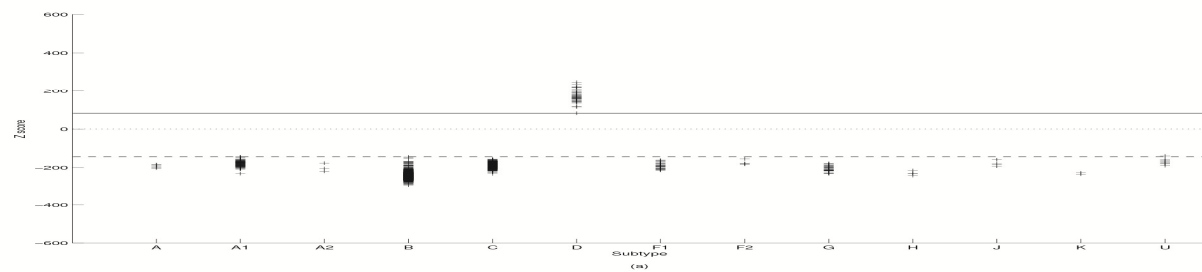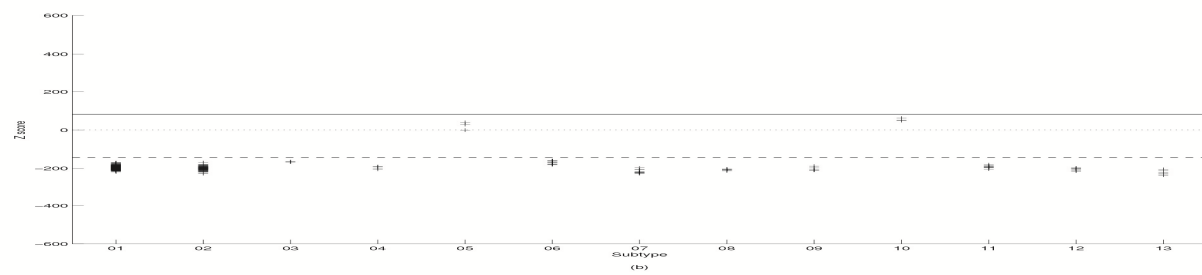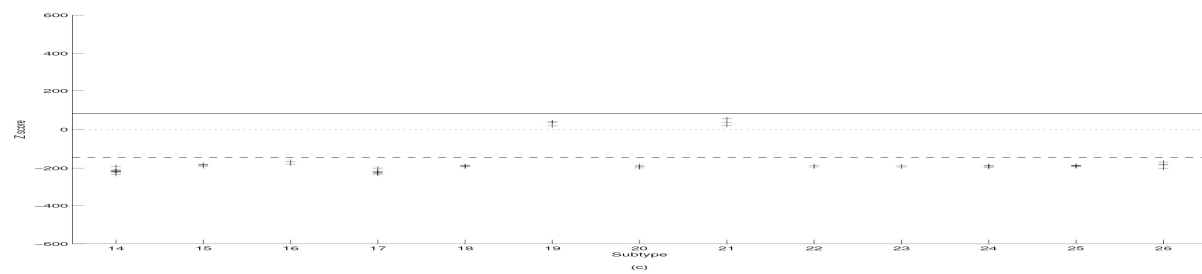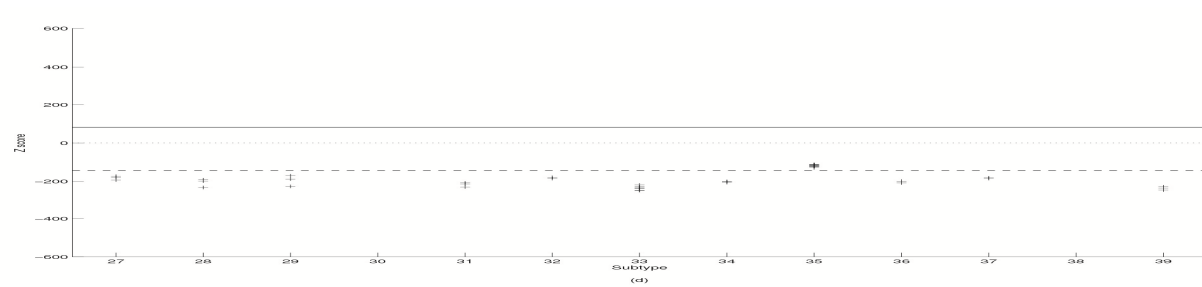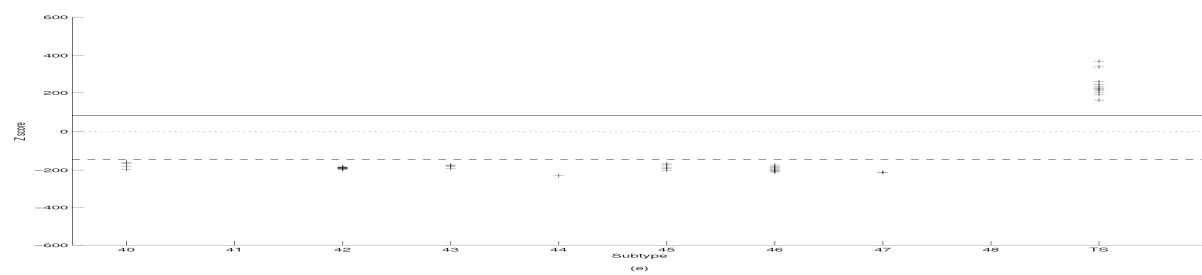

Supplement: Figure S8 — Detection of subtype D in the gag-pol region of CRF’s. The figures show the distribution of Z-scores of (a) pure sequences and (b)-(e) all CRF strains when the positive training set is constructed using fourteen sequences belonging to D. The solid and dashed lines correspond to the thresholds Tp and Tn respectively. Subtype D is not detected only in CRF16_A2D since very small fragments of D are present in the gag-pol region. There are no data points corresponding to CRF41_CD since sequences belonging to that strain are not yet publicly available. (PDF) [file pone.0036566.s008.pdf]

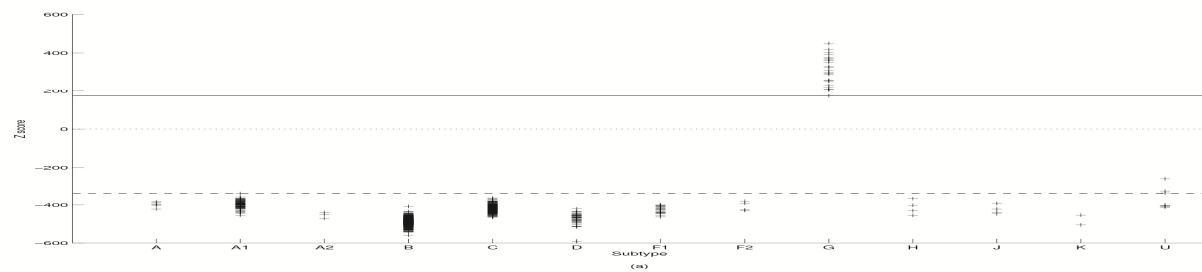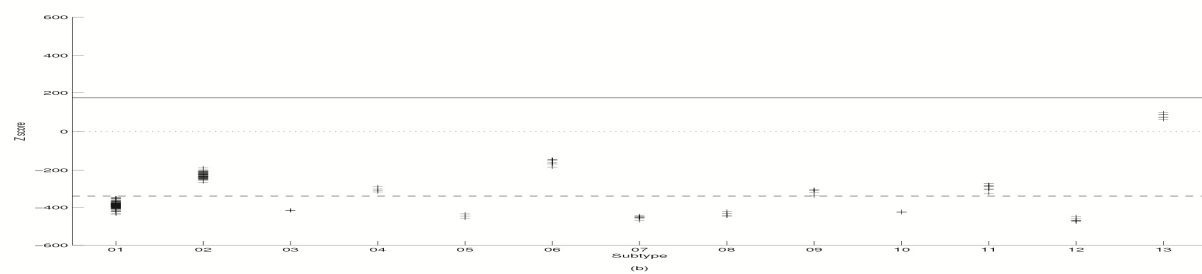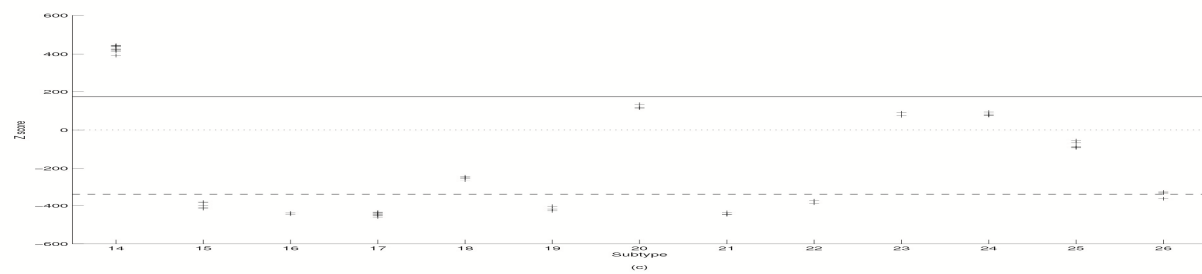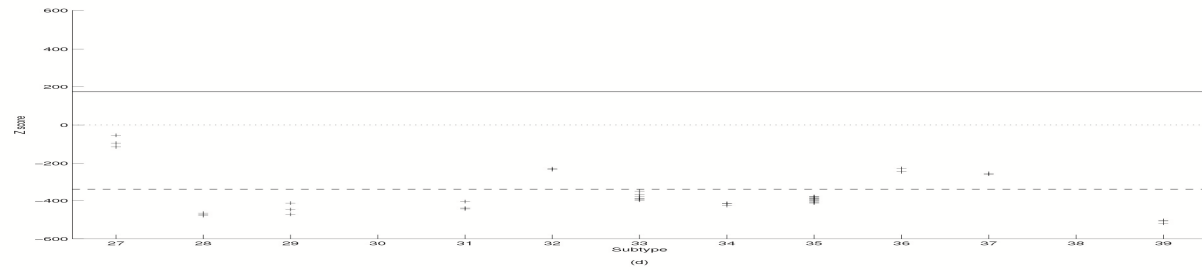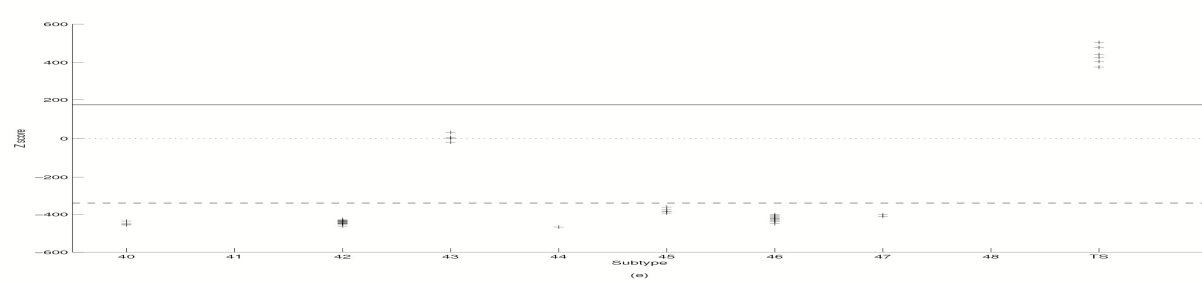

Supplement: Figure S9 — Detection of subtype G in the gag-pol region of CRF’s. The figures show the distribution of Z-scores of (a) pure sequences and (b)-(e) all CRF strains when the positive training set is constructed using six sequences belonging to G. The solid and dashed lines correspond to the thresholds Tp and Tn respectively. (PDF) [file pone.0036566.s009.pdf]

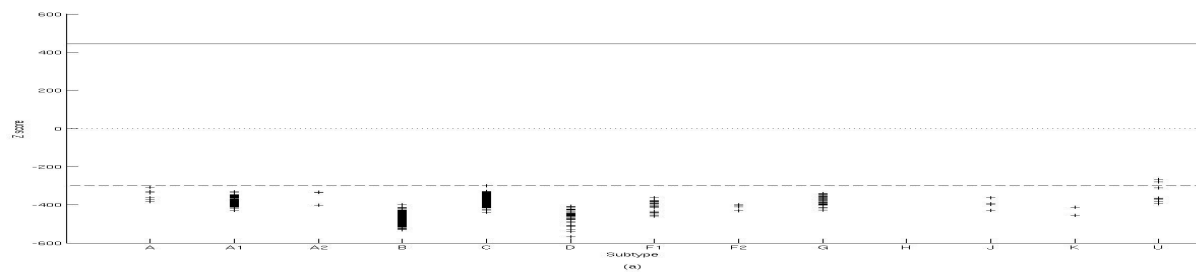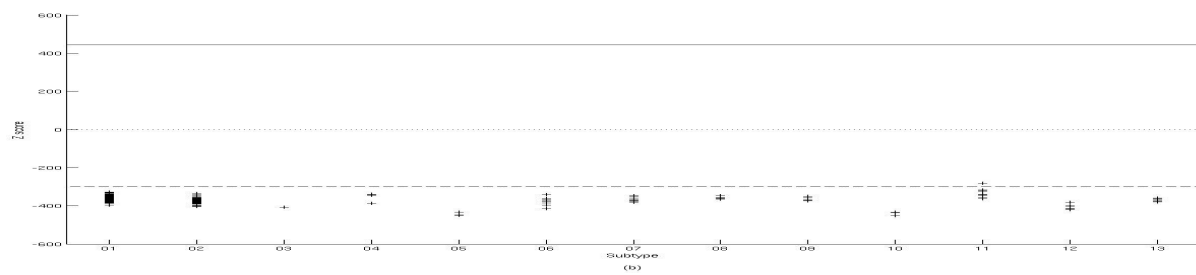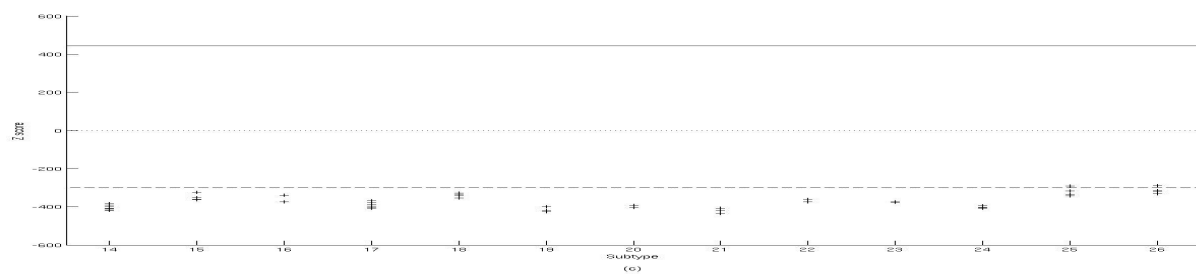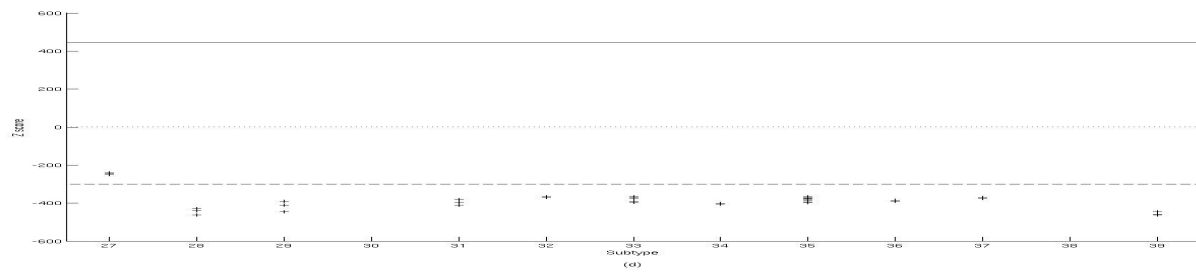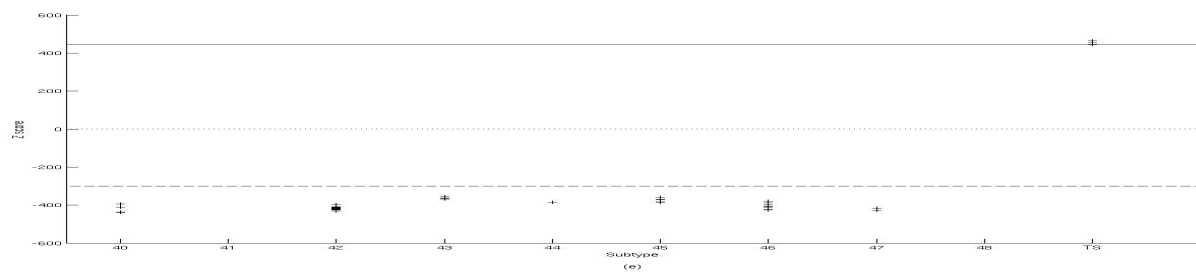

Supplement: Figure S10 — Detection of subtype H in the gag-pol region of CRF’s. The figures show the distribution of Z-scores of (a) pure sequences and (b)-(e) all CRF strains when the positive training set is constructed using four sequences belonging to H. The solid and dashed lines correspond to the thresholds Tp and Tn respectively. (PDF) [file pone.0036566.s010.pdf]

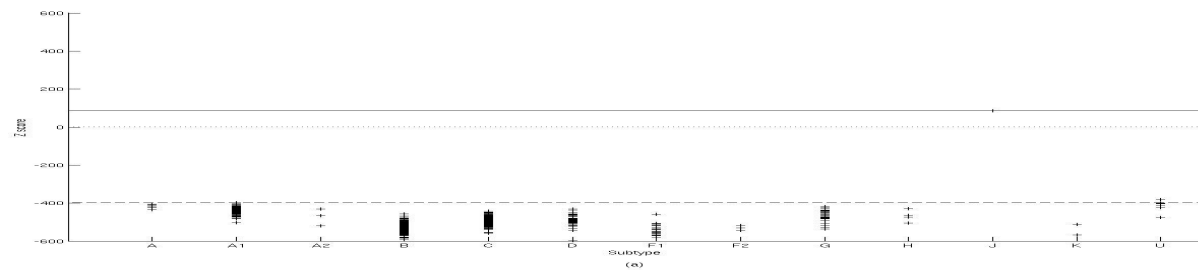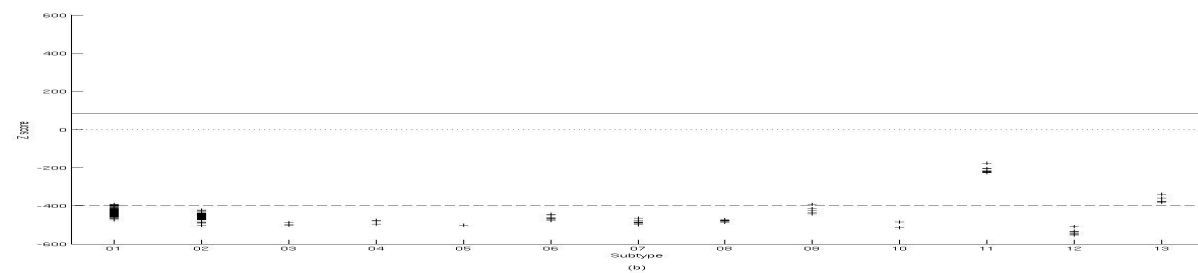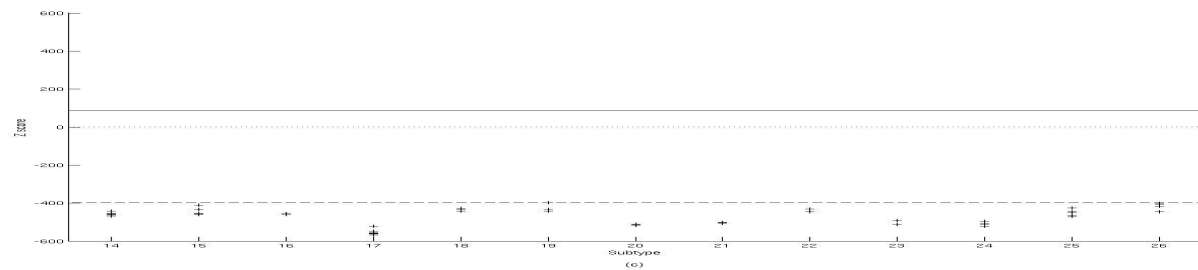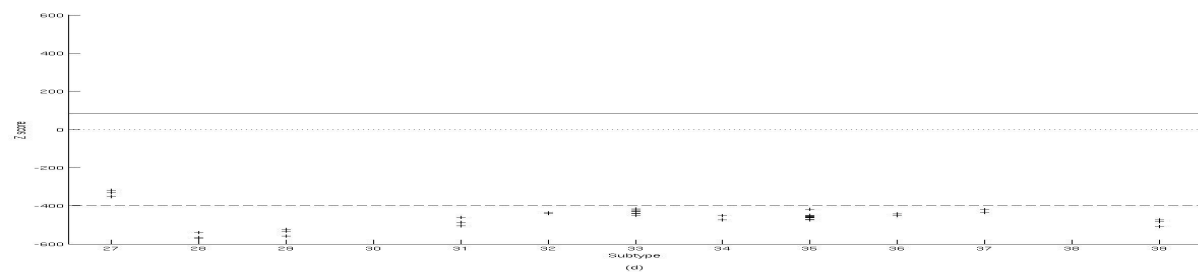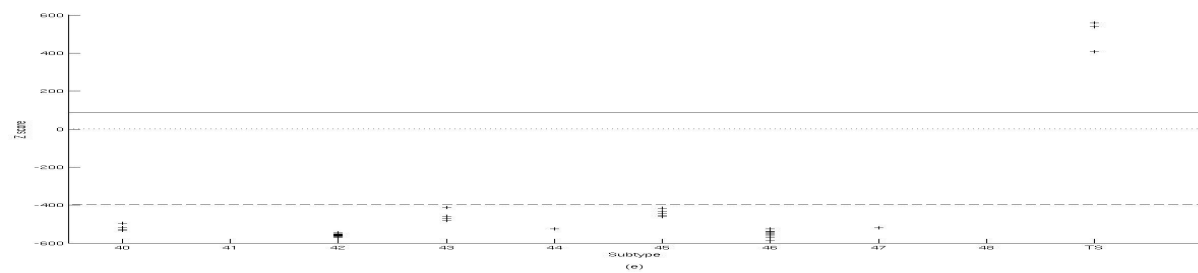

Supplement: Figure S11 — Detection of subtype J in the gag-pol region of CRF’s. The figures show the distribution of Z-scores of (a) pure sequences and (b)-(e) all CRF strains when the positive training set is constructed using three sequences belonging to J. The solid and dashed lines correspond to the thresholds Tp and Tn respectively. (PDF) [file pone.0036566.s011.pdf]

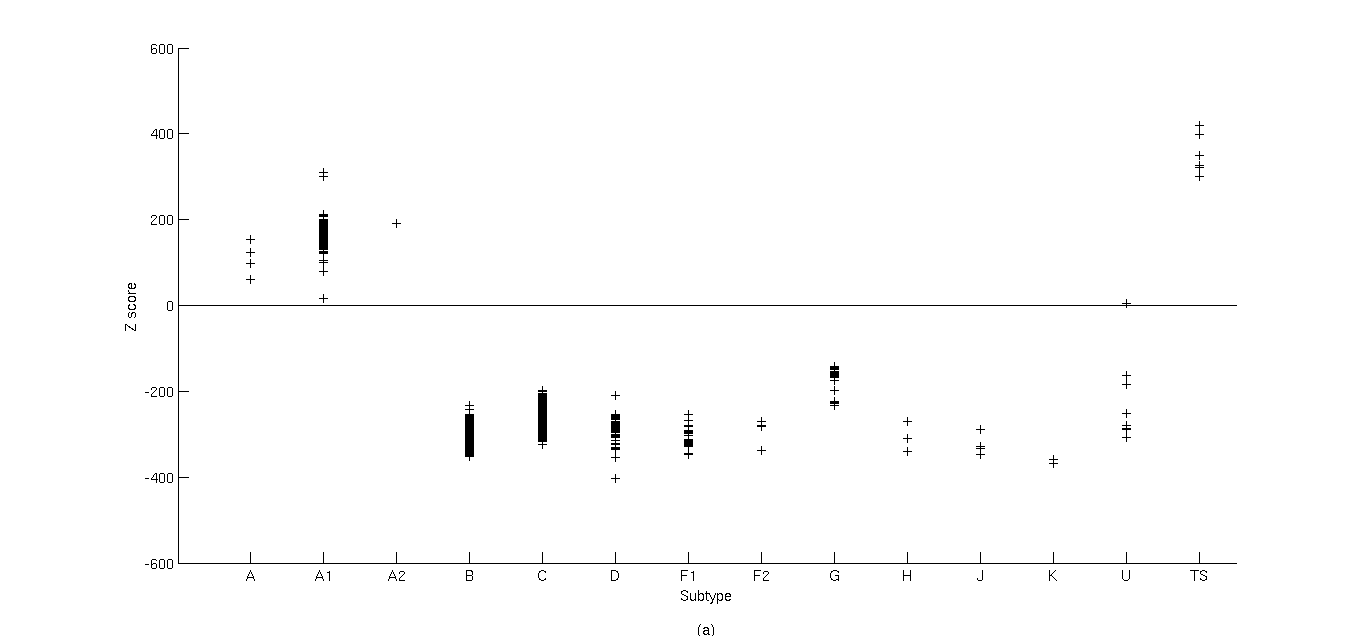

Supplement: Figure S12 — Subtype A classification using the improved method when the env region is used to construct the positive and negative pHMMs. Distribution of Z-scores for group M sequences. (TIF) [file pone.0036566.s012.tif]

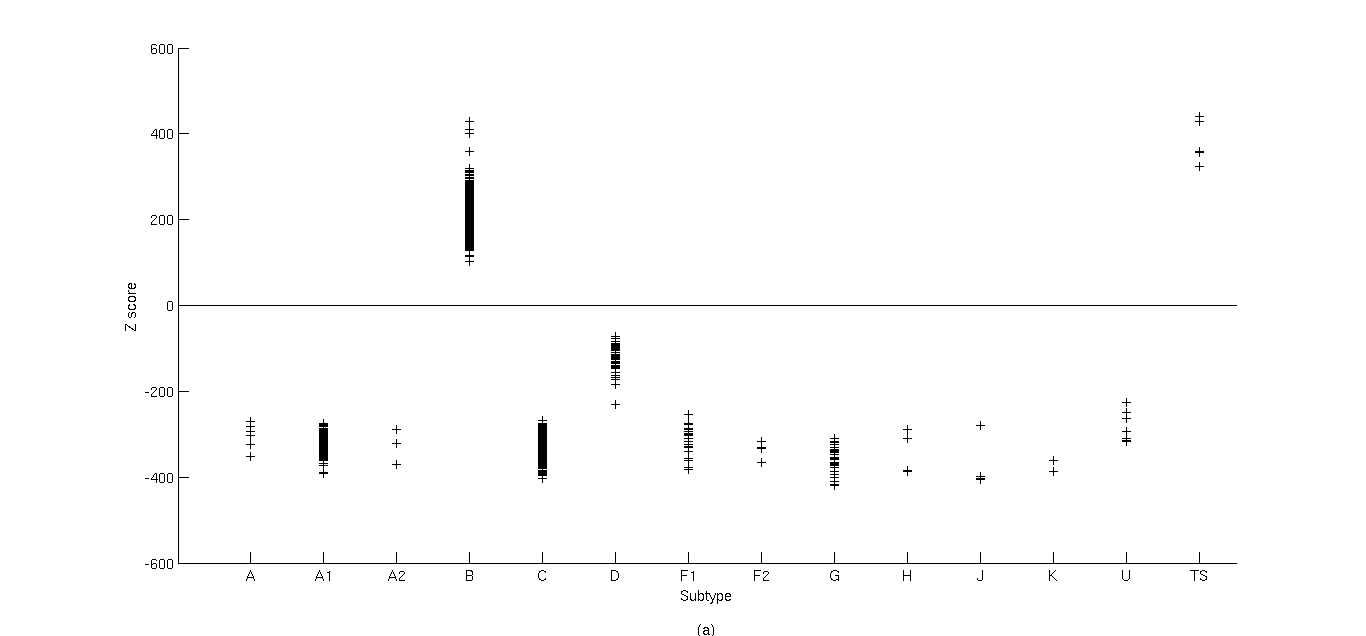

Supplement: Figure S13 — Subtype B classification using the improved method when the env region is used to construct the positive and negative pHMMs. Distribution of Z-scores for group M sequences. (TIF) [file pone.0036566.s013.tif]

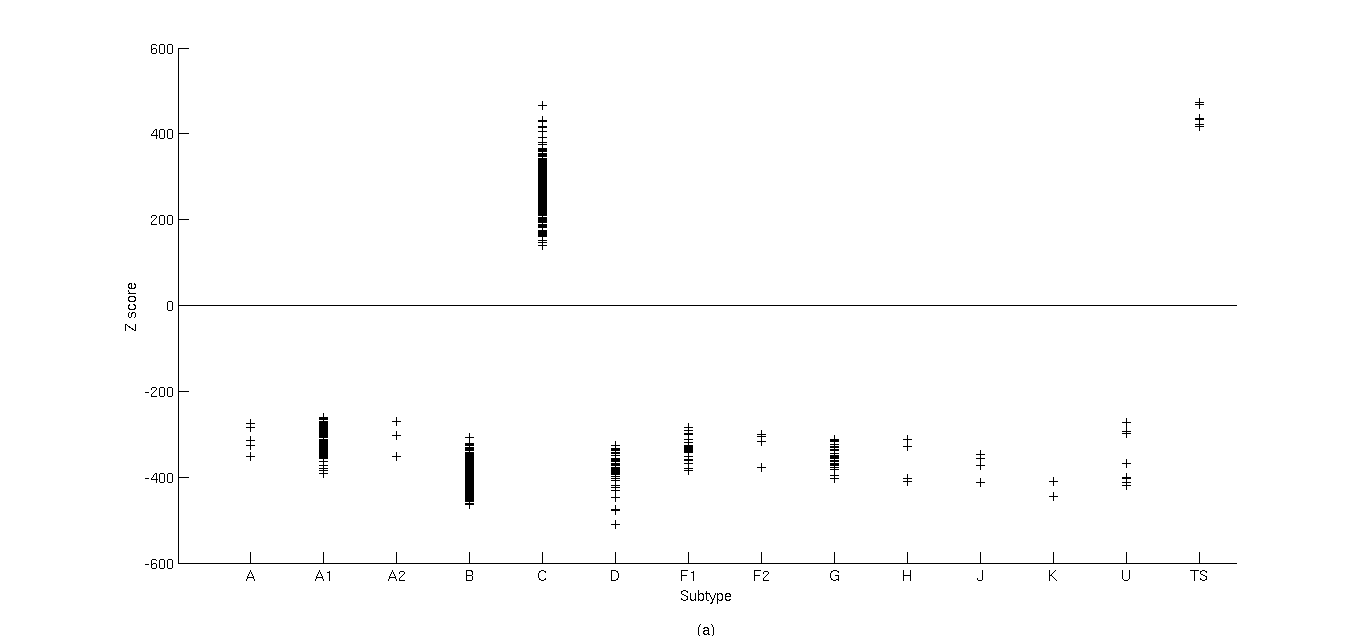

Supplement: Figure S14 — Subtype C classification using the improved method when the env region is used to construct the positive and negative pHMMs. Distribution of Z-scores for group M sequences. (TIF) [file pone.0036566.s014.tif]

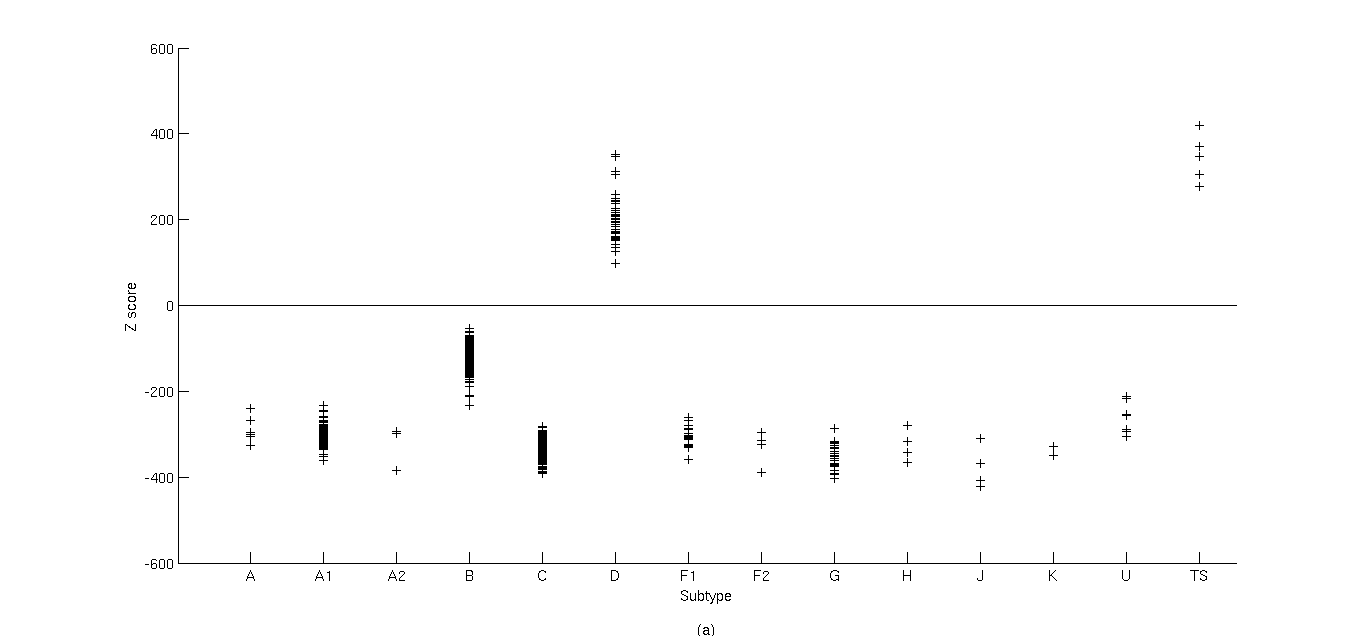

Supplement: Figure S15 — Subtype D classification using the improved method when the env region is used to construct the positive and negative pHMMs. Distribution of Z-scores for group M sequences. (TIF) [file pone.0036566.s015.tif]

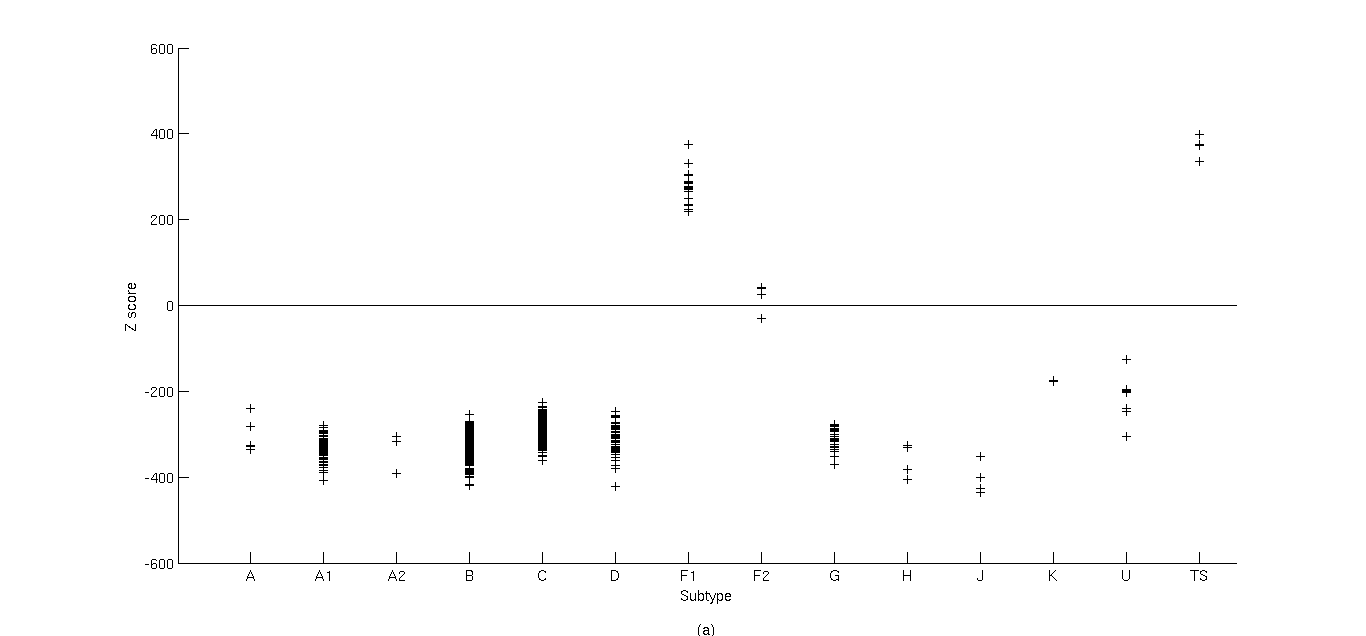

Supplement: Figure S16 — Subtype F1 classification using the improved method when the env region is used to construct the positive and negative pHMMs. Distribution of Z-scores for group M sequences. (TIF) [file pone.0036566.s016.tif]

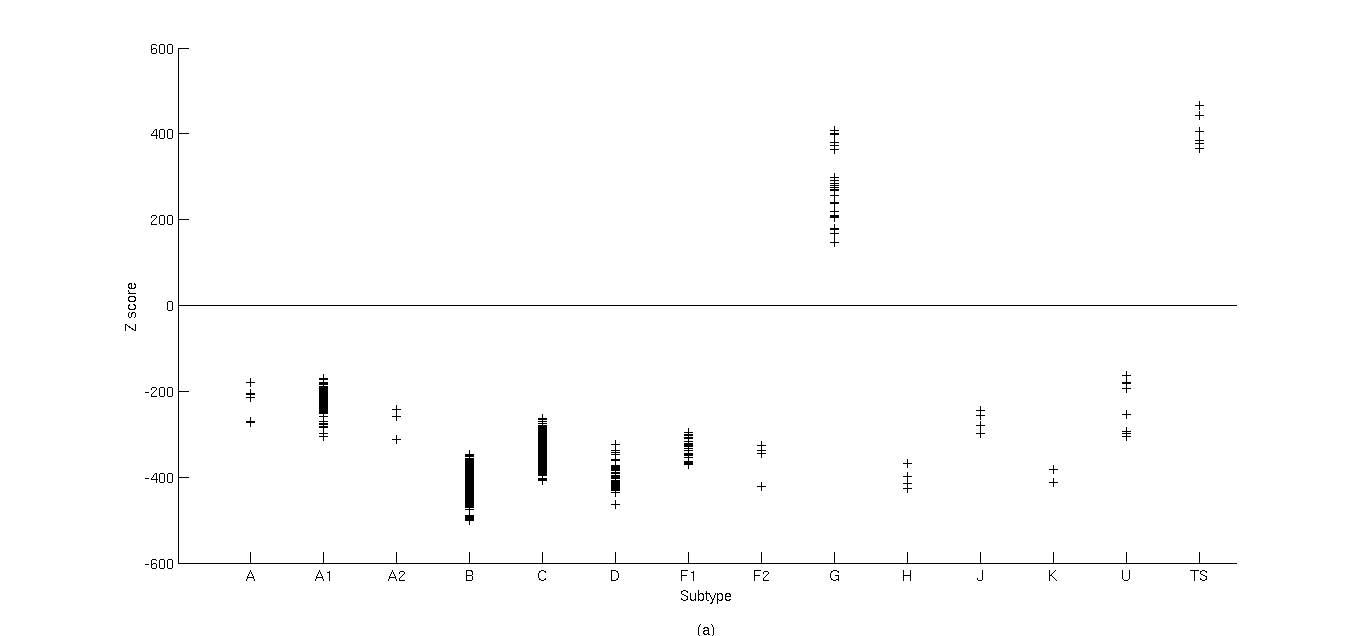

Supplement: Figure S17 — Subtype G classification using the improved method when the env region is used to construct the positive and negative pHMMs. Distribution of Z-scores for group M sequences. (TIF) [file pone.0036566.s017.tif]

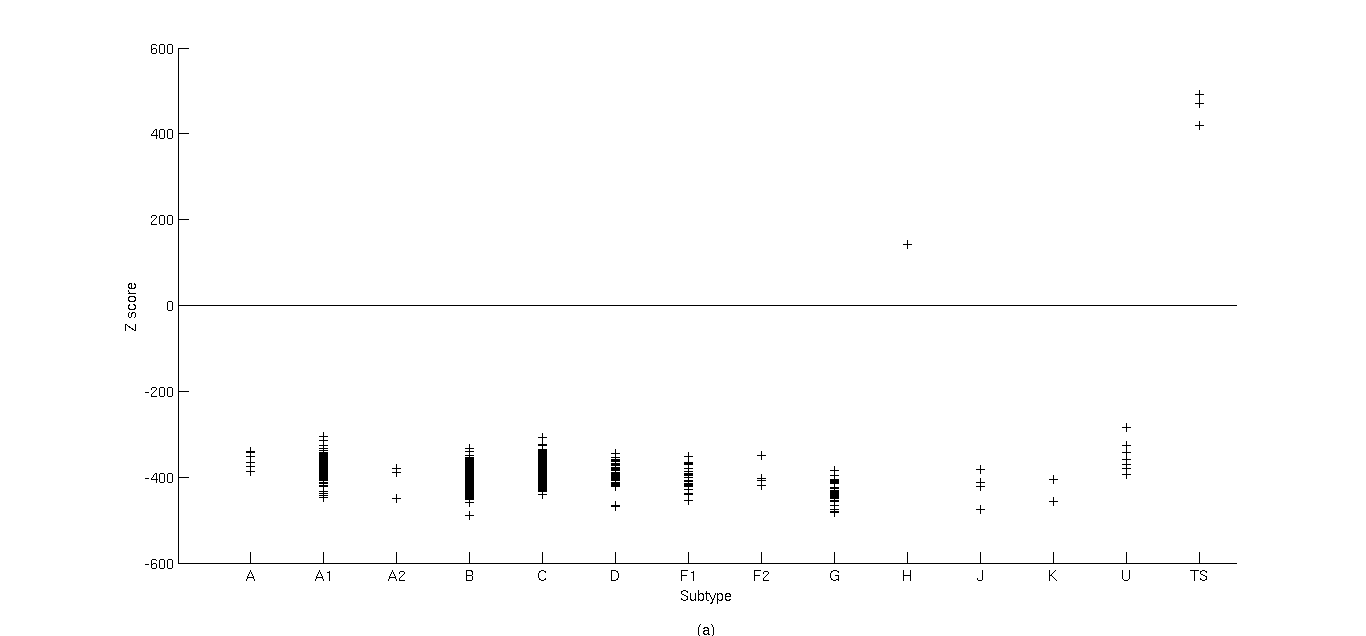

Supplement: Figure S18 — Subtype H classification using the improved method when the env region is used to construct the positive and negative pHMMs. Distribution of Z-scores for group M sequences. (TIF) [file pone.0036566.s018.tif]

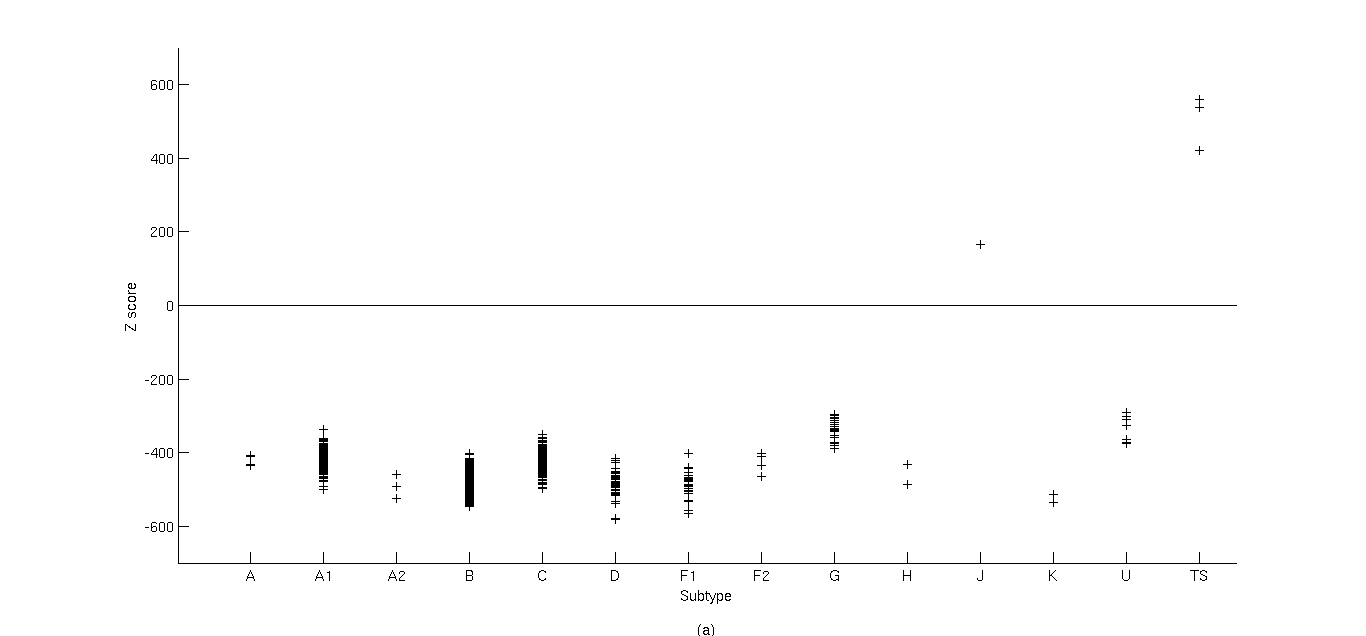

Supplement: Figure S19 — Subtype J classification using the improved method when the env region is used to construct the positive and negative pHMMs. Distribution of Z-scores for group M sequences. (TIF) [file pone.0036566.s019.tif]

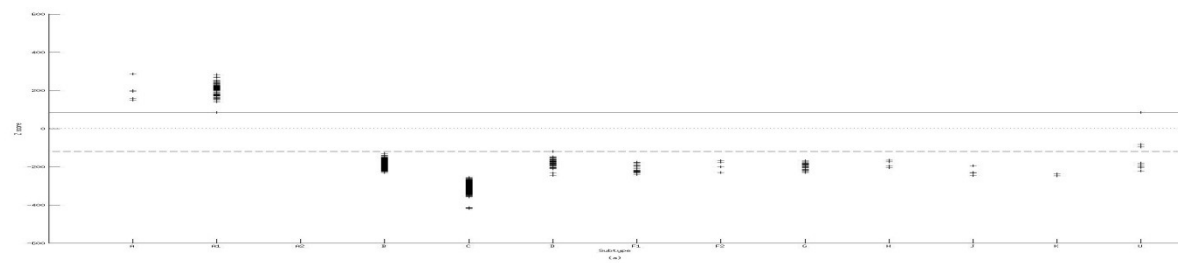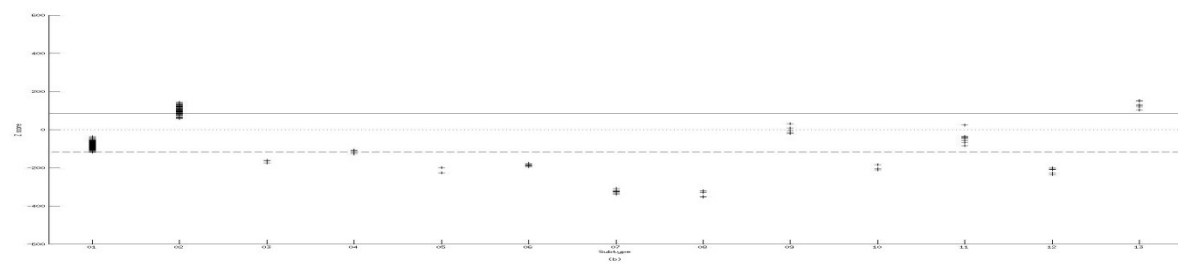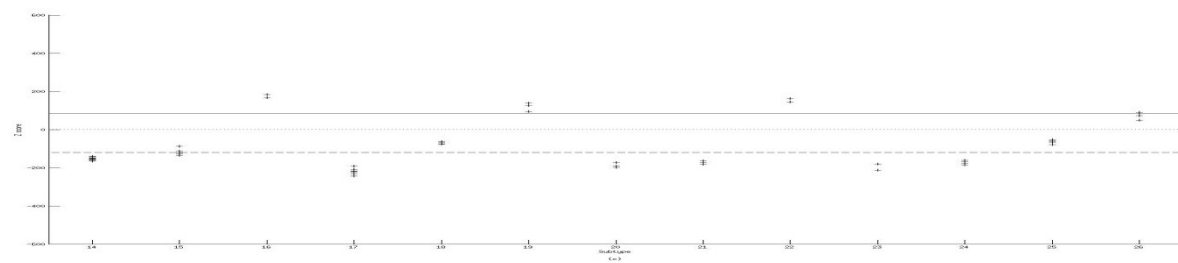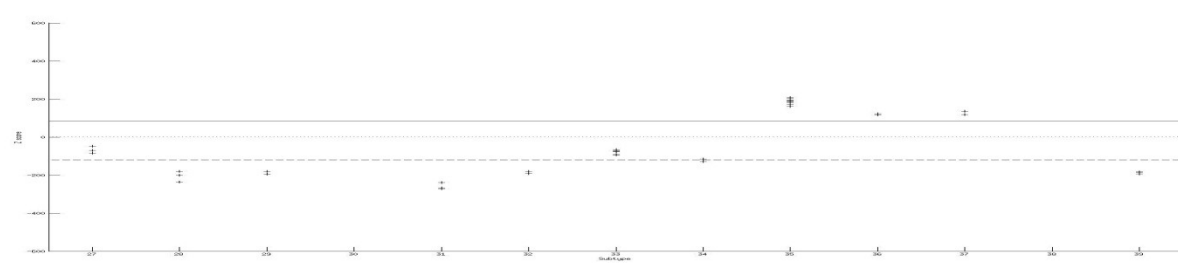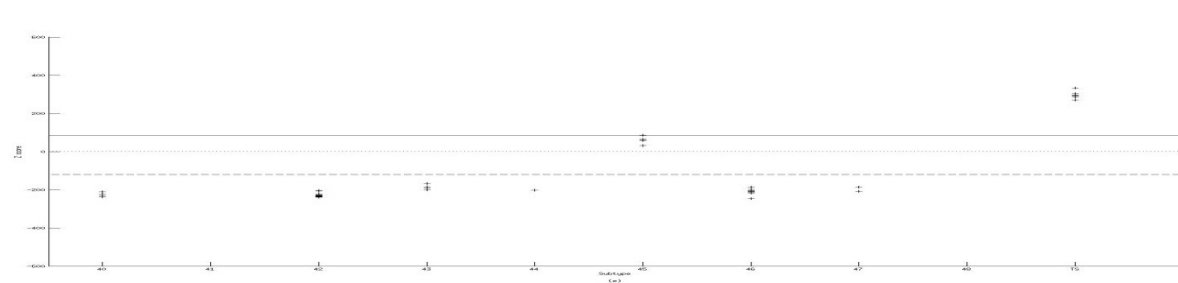

Supplement: Figure S20 — Detection of subtype A in the env region of CRF’s. The figures show the distribution of Z-scores of (a) pure sequences and (b)-(e): all CRF strains when the positive training set is constructed using a total of twelve sequences belonging to A,A1 and A2. The solid and dashed lines correspond to the thresholds Tp and Tn respectively. (PDF) [file pone.0036566.s020.pdf]

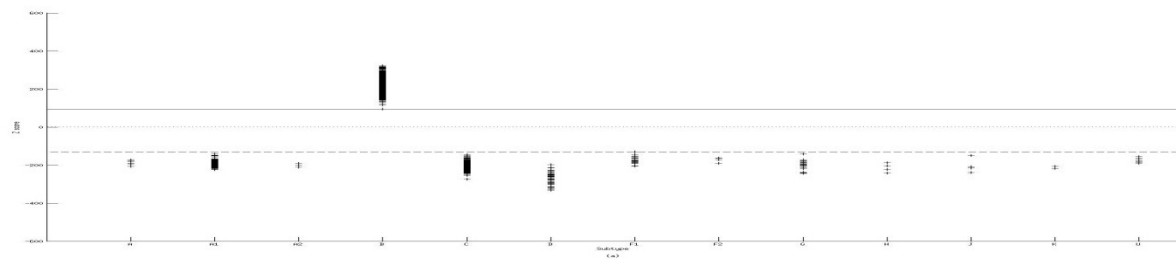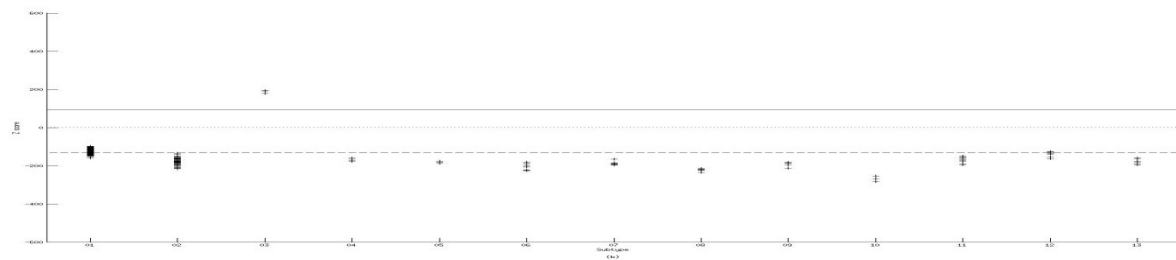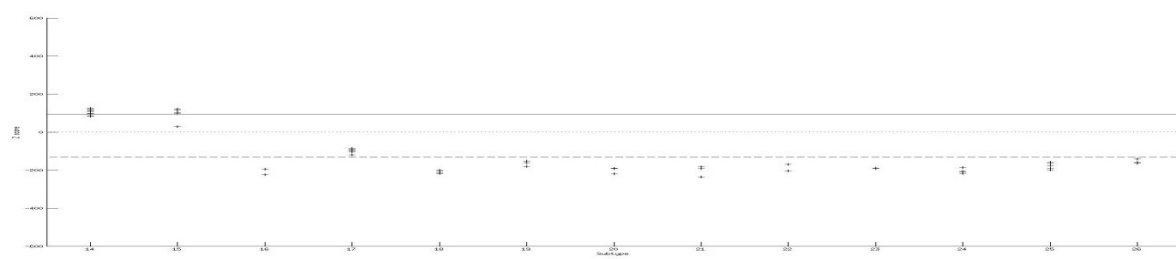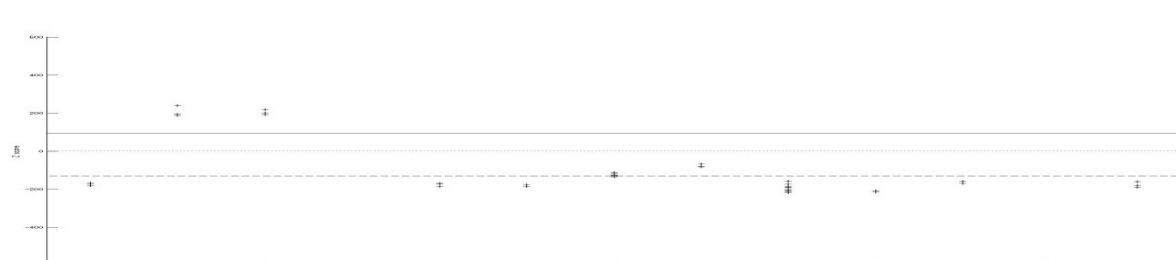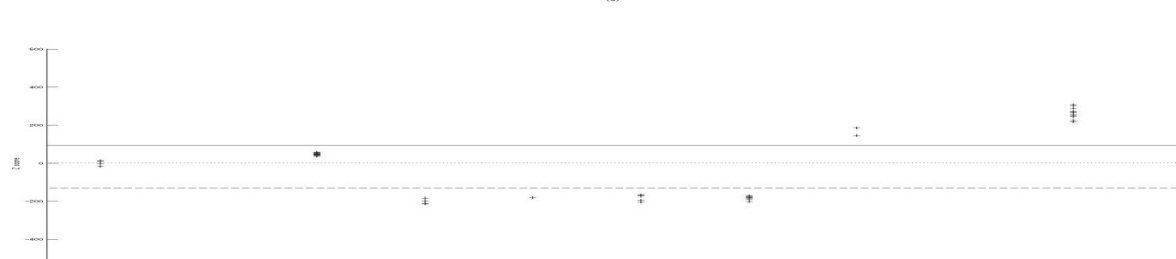

Supplement: Figure S21 — Detection of subtype B in the env region of CRF’s. The figures show the distribution of Z-scores of (a) pure sequences and (b)-(e): all CRF strains when the positive training set is constructed using thirty sequences each belonging to B. The solid and dashed lines correspond to the thresholds Tp and Tn respectively. (PDF) [file pone.0036566.s021.pdf]

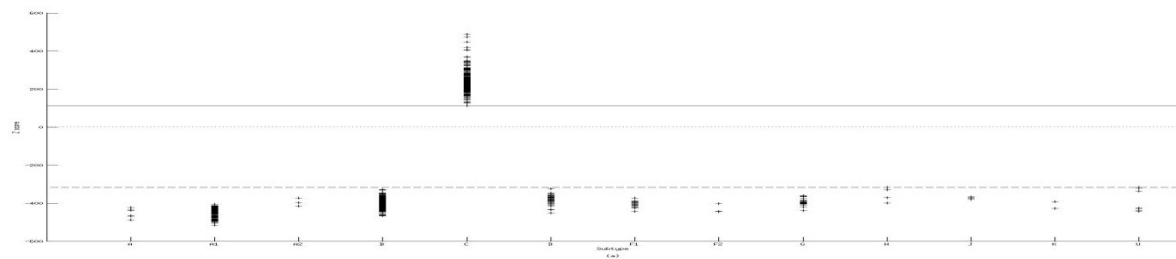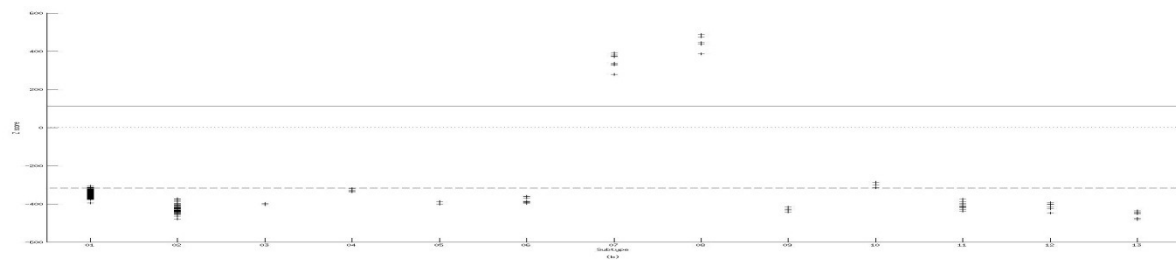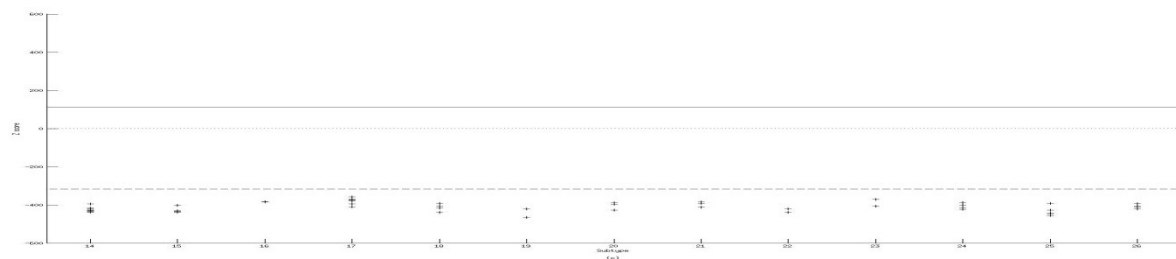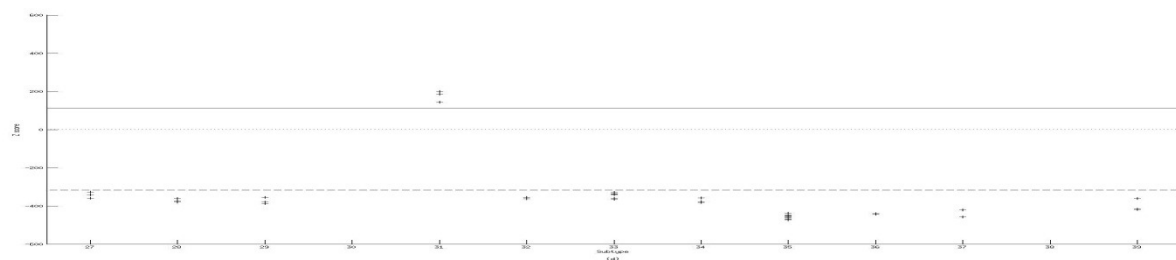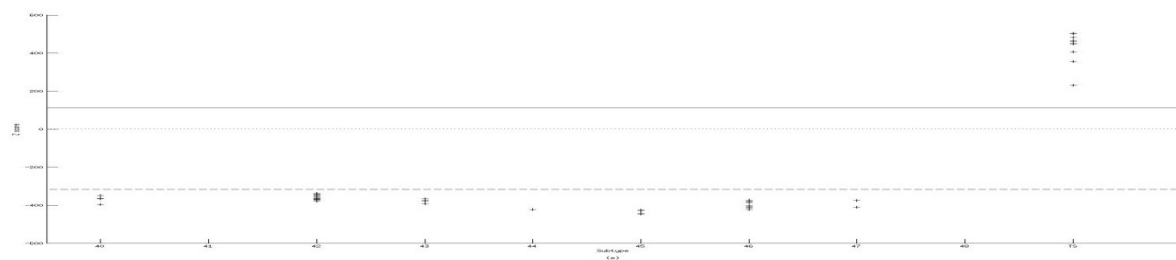

Supplement: Figure S22 — Detection of subtype C in the env region of CRF’s. The figures show the distribution of Z-scores of (a) pure sequences and (b)-(e) all CRF strains when the positive training set is constructed using ten sequences belonging to C. The solid and dashed lines correspond to the thresholds Tp and Tn respectively. (PDF) [file pone.0036566.s022.pdf]

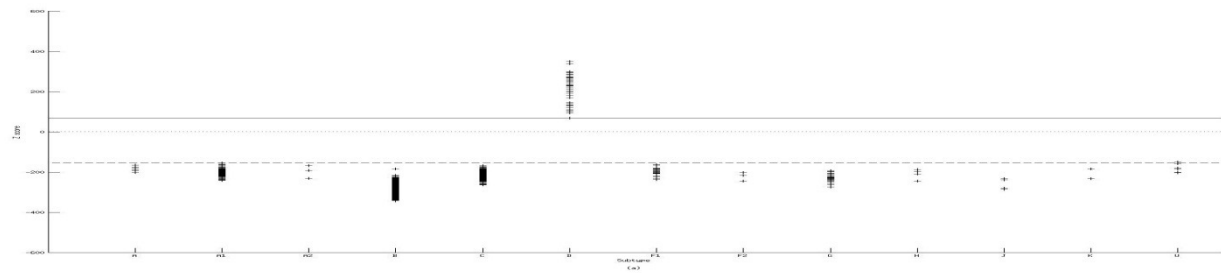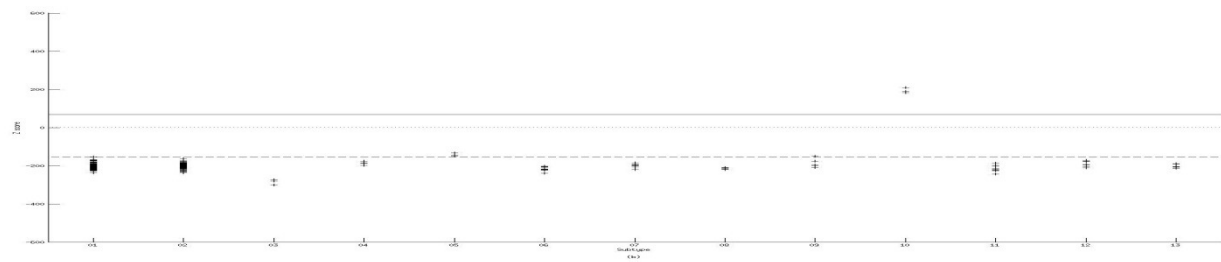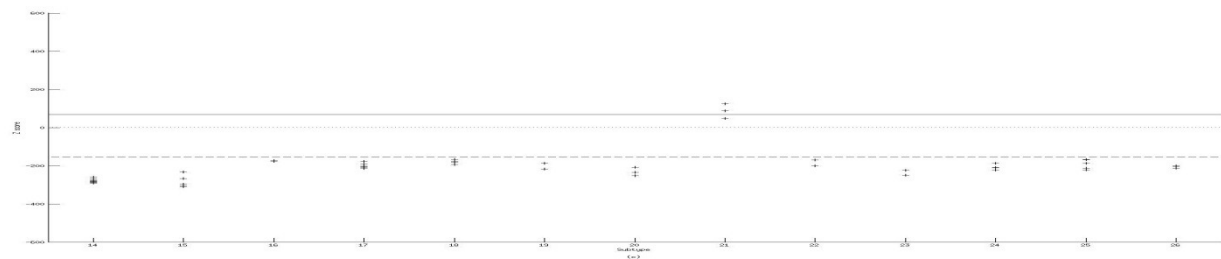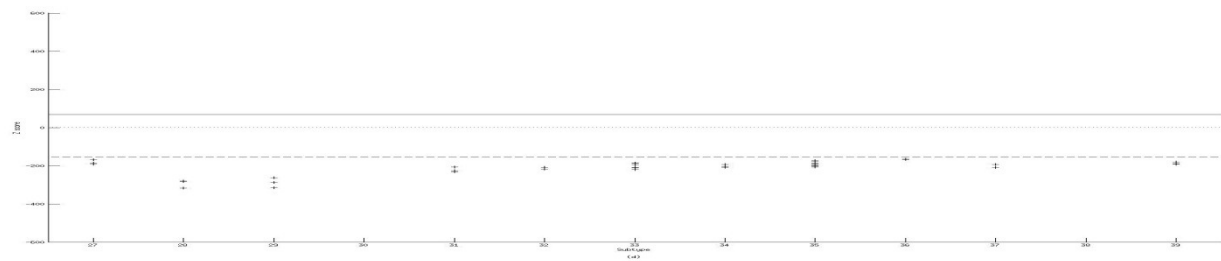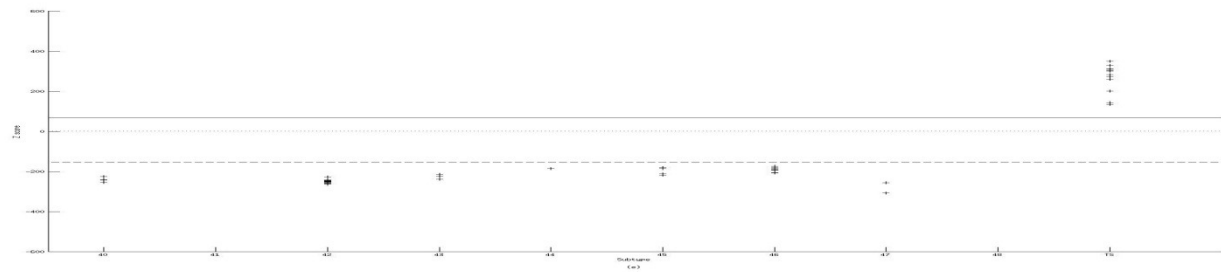

Supplement: Figure S23 — Detection of subtype D in the env region of CRF’s. The figures show the distribution of Z-scores of (a) pure sequences and (b)-(e) all CRF strains when the positive training set is constructed using twenty sequences belonging to D. The solid and dashed lines correspond to the thresholds Tp and Tn respectively. (PDF) [file pone.0036566.s023.pdf]

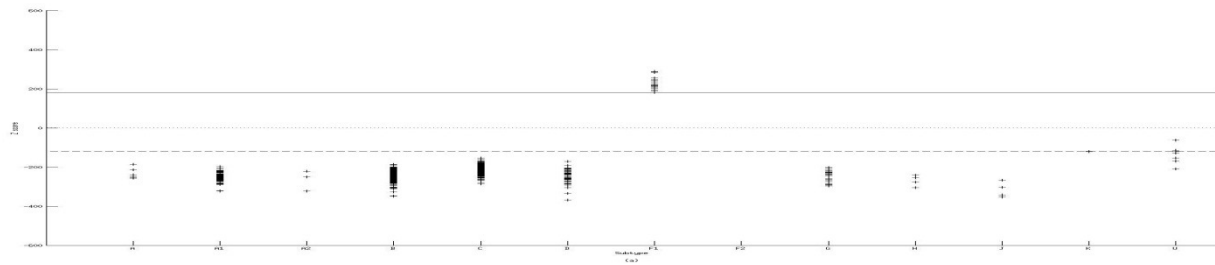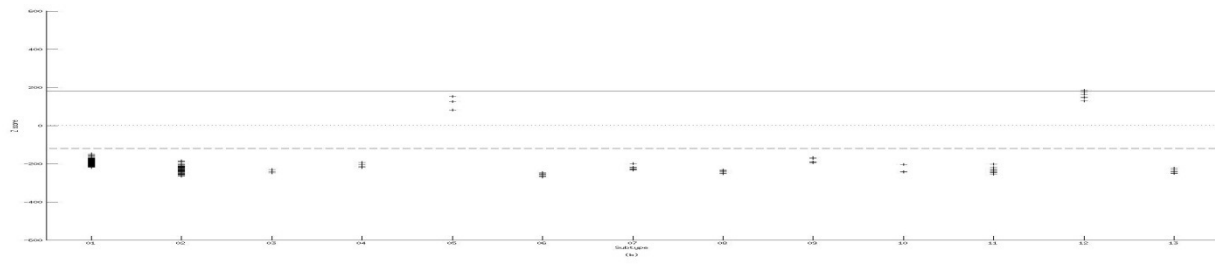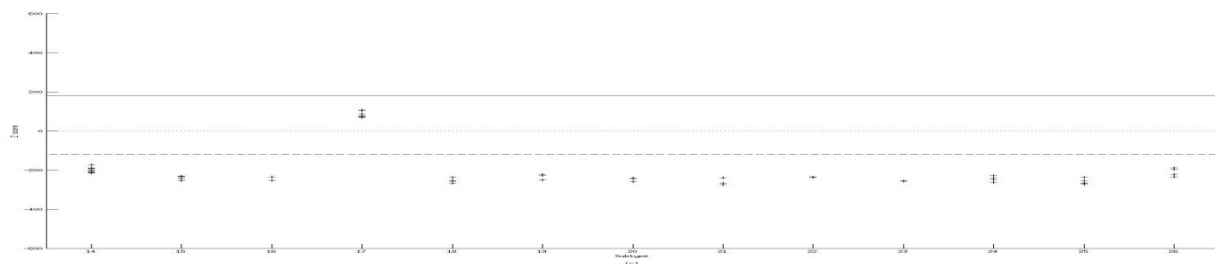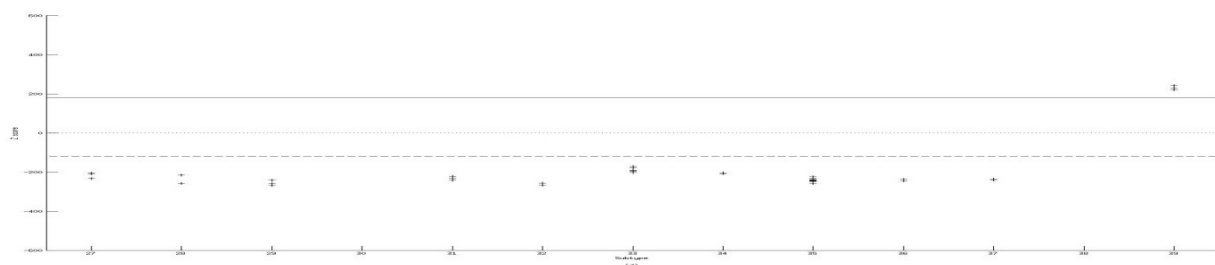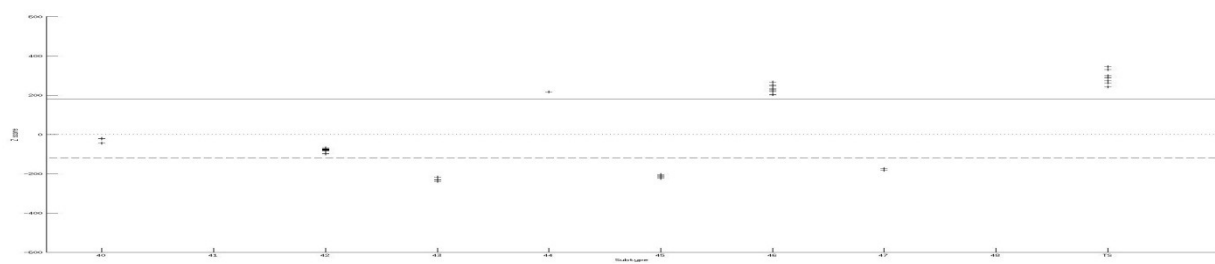

Supplement: Figure S24 — Detection of subtype F in the env region of CRF’s. The figures show the distribution of Z-scores of (a) pure sequences and (b)-(e) all CRF strains when the positive training set is constructed using twelve sequences belonging to F. The solid and dashed lines correspond to the thresholds Tp and Tn respectively. (PDF) [file pone.0036566.s024.pdf]

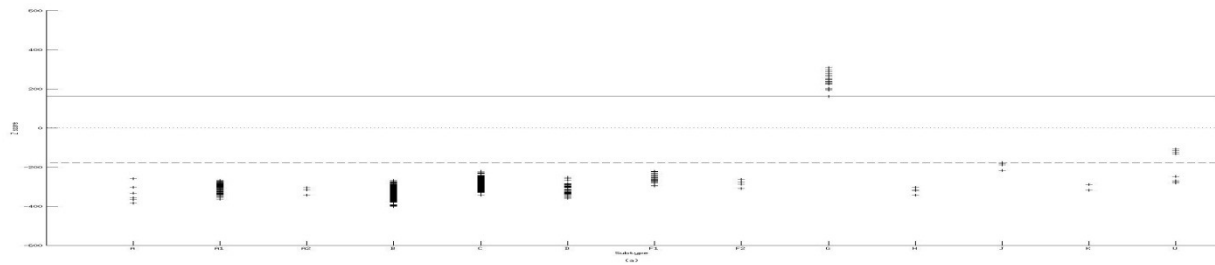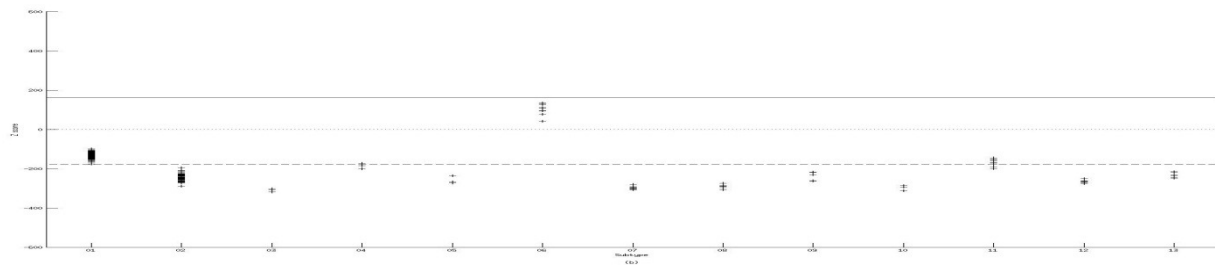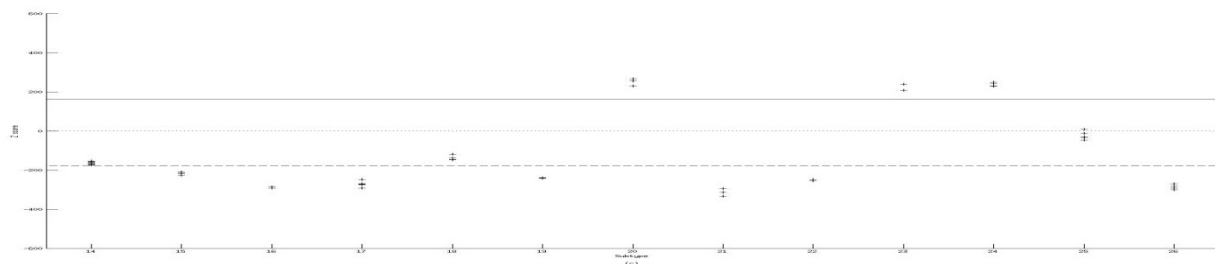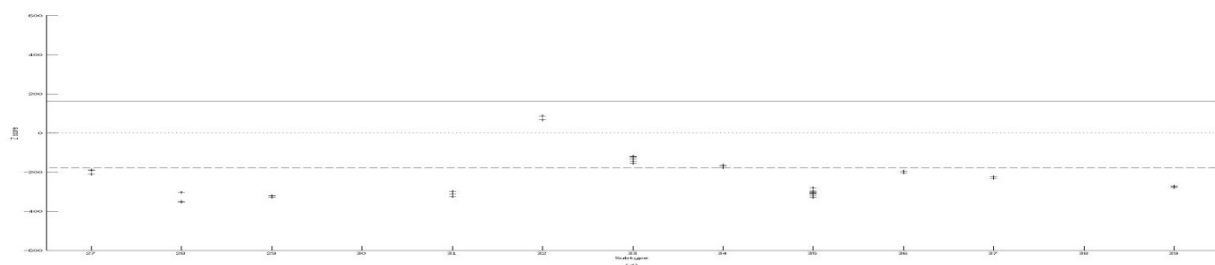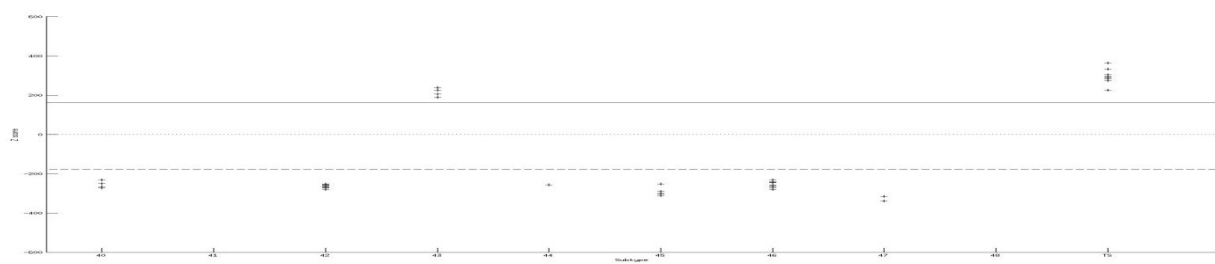

Supplement: Figure S25 — Detection of subtype G in the env region of CRF’s. The figures show the distribution of Z-scores of (a) pure sequences and (b)-(e) all CRF strains when the positive training set is constructed using twelve sequences belonging to G. The solid and dashed lines correspond to the thresholds Tp and Tn respectively. (PDF) [file pone.0036566.s025.pdf]

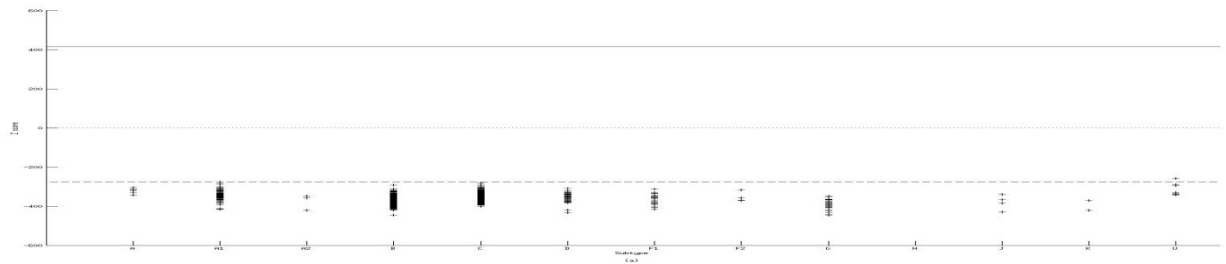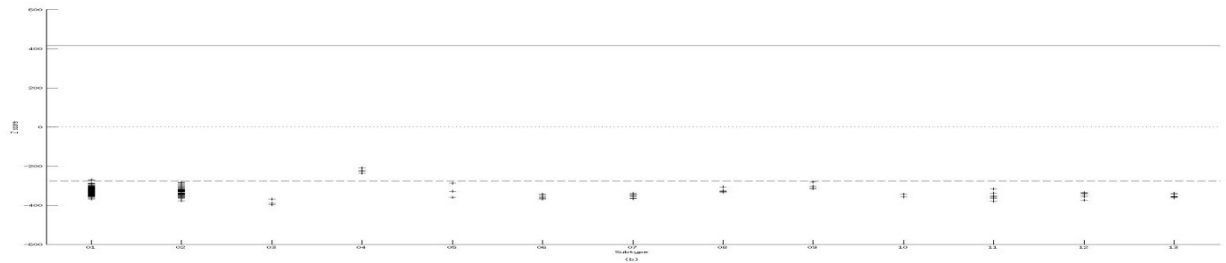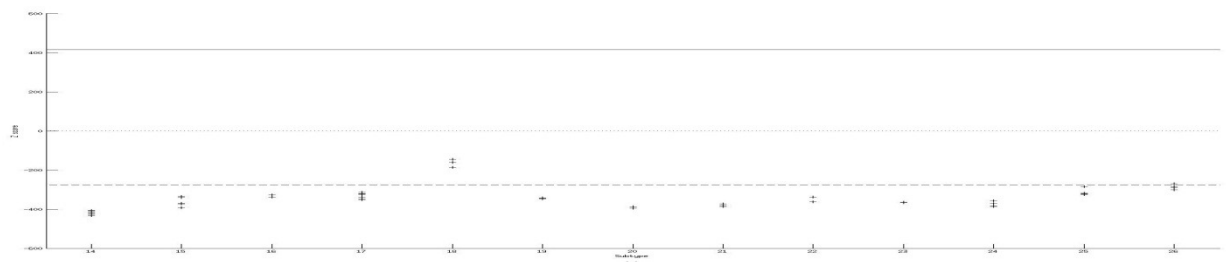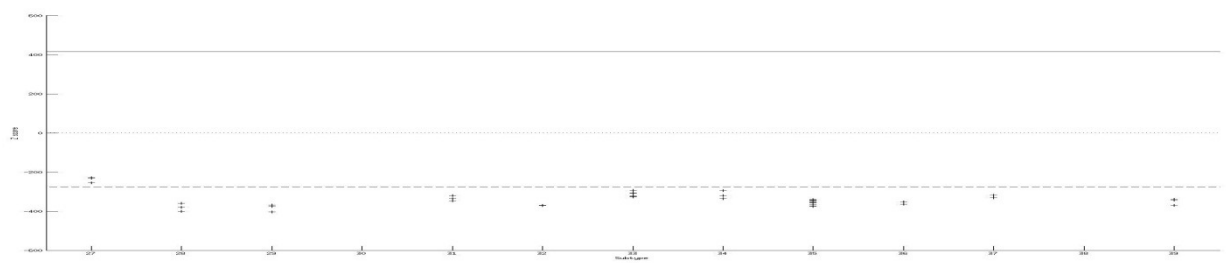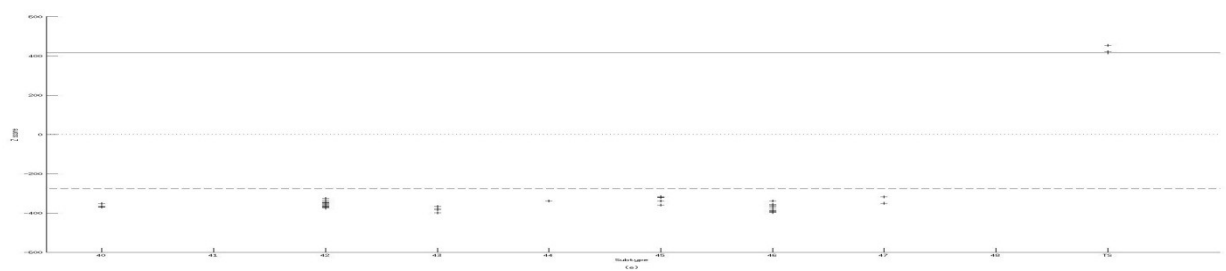

Supplement: Figure S26 — Detection of subtype H in the env region of CRF’s. The figures show the distribution of Z-scores of (a) pure sequences and (b)-(e) all CRF strains when the positive training set is constructed using four sequences belonging to H. The solid and dashed lines correspond to the thresholds Tp and Tn respectively. (PDF) [file pone.0036566.s026.pdf]

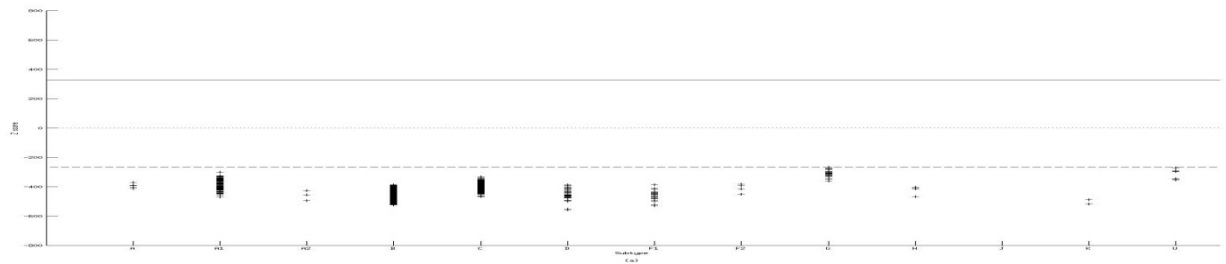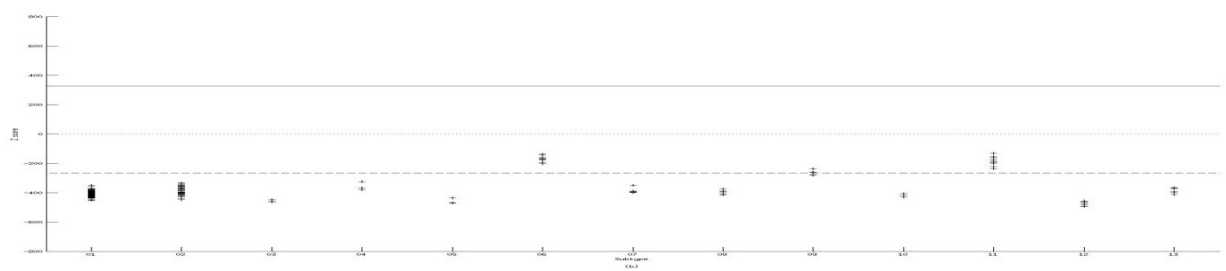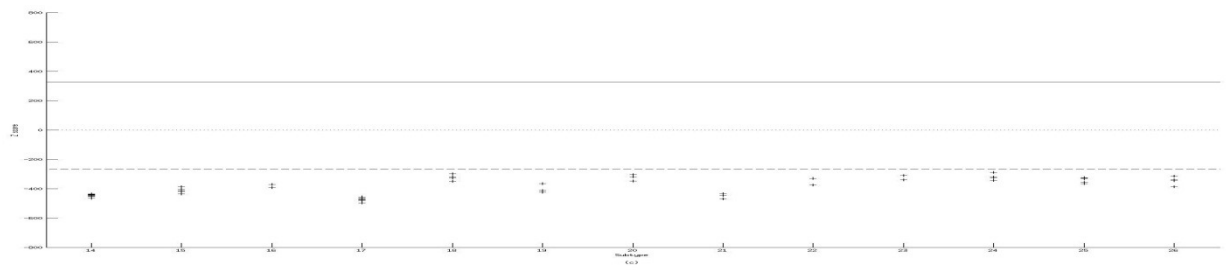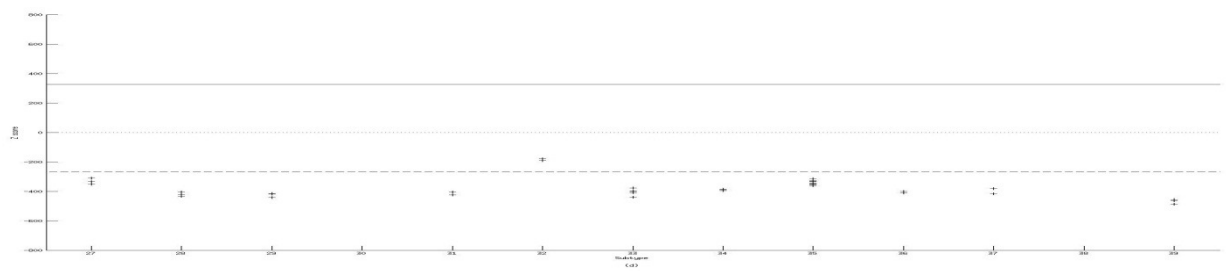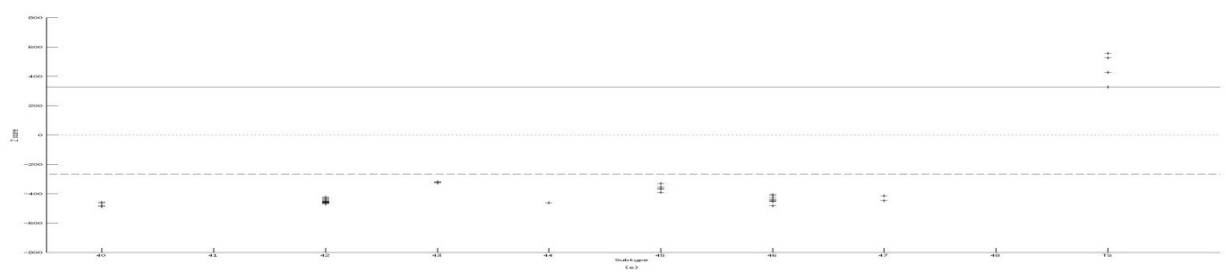

Supplement: Figure S27 — Detection of subtype J in the env region of CRF’s. The figures show the distribution of Z-scores of (a) pure sequences and (b)-(e) all CRF strains when the positive training set is constructed using four sequences belonging to J. The solid and dashed lines correspond to the thresholds Tp and Tn respectively. (PDF) [file pone.0036566.s027.pdf]
