# Supplementary material for: Classification of HIV-1 Sequences Using Profile Hidden Markov Models
Source: PLoS One. 2012 May 18;7(5):e36566. doi: 10.1371/journal.pone.0036566 (PMC3356369; doi:10.1371/journal.pone.0036566)
Supplement: Table S1 — Accession numbers of sequences making up the positive training set for all sub-types except H & J when the gag-pol region is used for classification. (PDF) [file pone.0036566.s028.pdf]

Table S1: Accession numbers of sequences making up the positive training set for all sub-types except H & J when the *gag-pol* region is used for classification.

A	B	C	D	F	G
AM000053	AB097870	AF110963	AY773340	GQ290462	AB287003
AM000054	AB286956	AF286228	DQ054367	DQ979025	FJ389364
AB098330	AB287370	AF110974	AB485650	AB485659	AB485663
AB253421	AB289589	AB254150	AJ519489	AJ249238	AY586548
AF286237	AB428560	AB097871	A14116	AB480300	AF423760
AF286238	AB480698	AB485645	U88822	DQ189088	AY612637
