# Supplementary material for: Classification of HIV-1 Sequences Using Profile Hidden Markov Models
Source: PLoS One. 2012 May 18;7(5):e36566. doi: 10.1371/journal.pone.0036566 (PMC3356369; doi:10.1371/journal.pone.0036566)
Supplement: Table S2 — Accession numbers of sequences making up the positive training set for sub-type H and J when the gag-pol region is used for classification. Since there are only 4 sequences of the gag-pol region for H and 3 for J, 3 of the 4 sequences of H and 2 of the 3 sequences of J were used to build the respective pHMMs which were then successful in classifying the remaining sequences. (PDF) [file pone.0036566.s029.pdf]

Table S2: Accession numbers of sequences making up the positive training set for sub-type H and J when the *gag-pol* region is used for classification. Since there are only 4 sequences of the *gag-pol* region for H and 3 for J, 3 of the 4 sequences of H and 2 of the 3 sequences of J were used to build the respective pHMMs which were then successful in classifying the remaining sequences.

**H**	**J**
AF005496	AF082394
AF190127	GU237072
AF190128	
