# Supplementary material for: Classification of HIV-1 Sequences Using Profile Hidden Markov Models
Source: PLoS One. 2012 May 18;7(5):e36566. doi: 10.1371/journal.pone.0036566 (PMC3356369; doi:10.1371/journal.pone.0036566)
Supplement: Table S4 — Accession numbers of sequences making up the negative training set for sub-type D when the gag-pol region is used for classification. (PDF) [file pone.0036566.s031.pdf]

Table S4: Accession numbers of sequences making up the negative training set for sub-type D when the *gag-pol* region is used for classification.

Subtype	Accession Number
A1	AB253422
A2	AF286238
B	AB565496
B	AF042102
B	AB480696
B	AF538305
B	AB485642
B	AF049495
C	AB254141
C	AB485645
F1	AB485656
F2	AJ249237
G	AB485662
G	AY586548
H	AF005496
H	FJ711703
J	AF082394
J	GU237072
K	AJ249235
K	AJ249239
