# Supplementary material for: Classification of HIV-1 Sequences Using Profile Hidden Markov Models
Source: PLoS One. 2012 May 18;7(5):e36566. doi: 10.1371/journal.pone.0036566 (PMC3356369; doi:10.1371/journal.pone.0036566)
Supplement: Table S5 — Number of sequences making up the positive training set used for determining whether a given sub-type (X) is present in the gag-pol region of a CRF. (PDF) [file pone.0036566.s032.pdf]

Table S5: Number of sequences making up the positive training set used for determining whether a given sub-type (X) is present in the *gag-pol* region of a CRF.

Subtype(X)	Number of Sequences	Accession Numbers
A	6	AM000053, AM000054, AB098330, AB253421, AF286237, AF286238
B	31	AB097870, AB221126, AB287364, AB287366, AB287371, AB289590, AB428557, AB480693, AB480697, AB564745, AB565498, AF004394, AF042101, AF049495, AF256205, AY173952, AY173956, AY275556, AY314045, AY314051, AY314058, AY331285, AY331297, AY423387, AY779552, AY818644, AY835754, AY835762, AY835771, AY839827, AB604949
C	16	AF286233, AF443087, AF110966, AY878056, AF443076, AF443094, AY585266, AF443111, AY228556, AY463227, AY043175, AY772693, AB254144, AB254153, AF110978, AF067154
D	14	A14116, A34828, AB485648, AB485650, AF133821, AJ488926, AJ519489, AY773338, AY773339, AY773340, EF633445, FJ388945, U88822, U88824
F	10	AB480298, AB485657, AB485658, AF075703, AF077336, DQ189088, DQ979023, DQ979024, AJ249236, AJ249237
G	6	AB287003, AB485663, AF423760, AY586548, AY612637, FJ389364
H	4	AF190127, FJ711703, AF005496, AF190128
J	3	GU237072, AF082394, AF082395
