# Supplementary material for: Classification of HIV-1 Sequences Using Profile Hidden Markov Models
Source: PLoS One. 2012 May 18;7(5):e36566. doi: 10.1371/journal.pone.0036566 (PMC3356369; doi:10.1371/journal.pone.0036566)
Supplement: Table S7 — Accession numbers of sequences making up the positive training set for all sub-types, except H and J, when the env region is used for classification. (PDF) [file pone.0036566.s034.pdf]

Table S7: Accession numbers of sequences making up the positive training set for all sub-types, except H and J, when the *env* region is used for classification.

A	B	C	D	F	G
AM000053	AB428560	AF286228	AY773340	GQ290462	AB287003
AM000054	AB287370	AB254150	AJ519489	AF075703	AF423760
AF286237	AB286956	AF110963	AB485650	DQ189088	AY612637
AF286238	AB480698	AB097871	DQ054367	AB485659	AY586548
AB098330	AB097870	AB485645	A14116	AJ249238	FJ389364
AB253421	AB289589	AF110974	U88822	DQ979025	AB485663
