# Supplementary material for: Classification of HIV-1 Sequences Using Profile Hidden Markov Models
Source: PLoS One. 2012 May 18;7(5):e36566. doi: 10.1371/journal.pone.0036566 (PMC3356369; doi:10.1371/journal.pone.0036566)
Supplement: Table S8 — Accession numbers of sequences making up the negative training set for all sub-types, except H and J, when the env region is used for classification. (PDF) [file pone.0036566.s035.pdf]

Table S8: Accession numbers of sequences making up the negative training set for all sub-types, except H and J, when the *env* region is used for classification.

Sub-type	Accession numbers
C	AB254141
C	AB485645
H	AF005496
H	FJ711703
G	AB485662
G	AY586548
A1	AB253422
A2	AF286238
D	A34828
D	AY773340
B	A04321
B	AB287372
K	AJ249235
K	AJ249239
F1	AB485656
F2	AJ249237
J	AF082394
J	GU237072
