# Supplementary material for: Classification of HIV-1 Sequences Using Profile Hidden Markov Models
Source: PLoS One. 2012 May 18;7(5):e36566. doi: 10.1371/journal.pone.0036566 (PMC3356369; doi:10.1371/journal.pone.0036566)
Supplement: Table S9 — Accession numbers of sequences making up the positive training set for sub-type H and J when the env region is used for classification. Since there are only 4 sequences of the env region for both H and J, three were used to build the pHMM which was then successful in classifying the remaining sequence. Note that a choice of any 3 of the 4 env sequences can be used to build a pHMM which can successfully classify the remaining one. (PDF) [file pone.0036566.s036.pdf]

Table S9: Accession numbers of sequences making up the positive training set for sub-type H and J when the *env* region is used for classification. Since there are only 4 sequences of the *env* region for both H and J, three were used to build the pHMM which was then successful in classifying the remaining sequence. Note that a choice of ***any*** 3 of the 4 *env* sequences can be used to build a pHMM which can successfully classify the remaining one.

**H**	**J**
AF190127	EF614151
AF005496	AF082394
AF190128	AF082395
