# Supplementary material for: Classification of HIV-1 Sequences Using Profile Hidden Markov Models
Source: PLoS One. 2012 May 18;7(5):e36566. doi: 10.1371/journal.pone.0036566 (PMC3356369; doi:10.1371/journal.pone.0036566)
Supplement: Table S12 — Thresholds for detection of sub-types in the gag-pol region of CRF strains. (PDF) [file pone.0036566.s039.pdf]

Table S12: Thresholds for detection of sub-types in the *gag-pol* region of CRF strains.

**Sub-Type**	**Tp**	**Tn**
A	119.7	-226.9
B	78.6	-195.1
C	104.4	-365.5
D	82.2	-144.2
F	63.9	-164.2
G	173.6	-340.6
H	443.1	-299.3
J	85.7	-398.0
