# Supplementary material for: Classification of HIV-1 Sequences Using Profile Hidden Markov Models
Source: PLoS One. 2012 May 18;7(5):e36566. doi: 10.1371/journal.pone.0036566 (PMC3356369; doi:10.1371/journal.pone.0036566)
Supplement: Table S13 — Number of sequences making up the positive training set used for determining whether a given sub-type (X) is present in the env region of a CRF. (PDF) [file pone.0036566.s040.pdf]

Table S13: Number of sequences making up the positive training set used for determining whether a given sub-type (X) is present in the *env* region of a CRF.

Subtype(X)	Number of Sequences
A	12
B	30
C	10
D	20
F	12
G	12
H	4
J	4
