# Supplementary material for: Classification of HIV-1 Sequences Using Profile Hidden Markov Models
Source: PLoS One. 2012 May 18;7(5):e36566. doi: 10.1371/journal.pone.0036566 (PMC3356369; doi:10.1371/journal.pone.0036566)
Supplement: Table S14 — Number of sequences of each sub-type making up the negative training set used for determining whether a given sub-type (X) is present in the env region of a CRF. (PDF) [file pone.0036566.s041.pdf]

Table S14: Number of sequences of each sub-type making up the negative training set used for determining whether a given sub-type (X) is present in the *env* region of a CRF.

**X=A**		**X=B**		**X=C**		**X=D**		**X=F**		**X=G**	
subtype	No.	subtype	No.	subtype	No.	subtype	No.	subtype	No.	subtype	No.
A	0	A	2	A	12	A	2	A	2	A	12
B	2	B	0	B	2	B	30	B	2	B	2
C	12	C	2	C	0	C	2	C	2	C	2
D	2	D	28	D	2	D	0	D	2	D	2
F	2	F	2	F	8	F	2	F	0	F	2
G	6	G	2	G	2	G	2	G	2	G	0
H	2	H	2	H	2	H	2	H	2	H	2
J	2	J	2	J	2	J	2	J	2	J	2
