# Supplementary material for: Classification of HIV-1 Sequences Using Profile Hidden Markov Models
Source: PLoS One. 2012 May 18;7(5):e36566. doi: 10.1371/journal.pone.0036566 (PMC3356369; doi:10.1371/journal.pone.0036566)
Supplement: Table S15 — Thresholds for detection of sub-types in the env region of CRF strains. (PDF) [file pone.0036566.s042.pdf]

**Table S15:** Thresholds for detection of sub-types in the *env* region of CRF strains.

| **Sub-Type** | **Tp** | **Tn** |
|---|---|---|
| A | 84.8 | -119.5 |
| B | 95.1 | -129.9 |
| C | 112.5 | -317.4 |
| D | 67.7 | -155.6 |
| F | 181.1 | -120.2 |
| G | 161.7 | -177.6 |
| H | 415.0 | -275.3 |
| J | 325.7 | -268.6 |
