# Supplementary material for: Classification of HIV-1 Sequences Using Profile Hidden Markov Models
Source: PLoS One. 2012 May 18;7(5):e36566. doi: 10.1371/journal.pone.0036566 (PMC3356369; doi:10.1371/journal.pone.0036566)
Supplement: File S1 — Instructions for classifying a query sequence (Q) using our pHMM method. (PDF) [file pone.0036566.s043.pdf]

Instructions for classifying a query sequence (Q) using our pHMM method

1. Take some sequences of the *gag-pol* coding region from the subtype X to determine if Q belongs to sub-type X. Example: six sequences of sub-type A are taken to build the positive profile HMM for A.

2. Create a Multiple Sequence Alignment (MSA) of these sequences using the MUSCLE package.

3. Build the profile HMM from the MSA obtained above by using the program *hmmbuild* of HMMER 3. This gives the positive profile HMM for subtype X.

4. Generate bit score (score_pos(Q)) for the query sequence using the positive profile HMM for sub-type X, by using the program *hmmsearch* of HMMER 3.

5. Take n sequences each of the *gag-pol* coding region for each of the subtypes which are different from X. Usually n=2 for all sub-types unless specified otherwise in the "supporting information" file.

6. Create a MSA of these sequences selected in point 5 using the MUSCLE package.

7. Build the profile HMM from the MSA obtained above by using the program *hmmbuild*. This gives the negative profile HMM for subtype X.

8. Generate bit score (score_neg(Q)) for the query sequence using the negative profile HMM for sub-type X by using program *hmmsearch* of HMMER3 .

9. Z score of the query sequence (Q) is defined as: Z-score(Q) = score_pos(Q) - score_neg(Q)

10 If Z-score(Q) is positive then Q belongs to subtype X, otherwise it belongs to some other sub-type.

11. Repeat process for other subtypes to accurately identify the sub-type to which the query sequence Q belongs.
